# A gH/gL-encoding replicon vaccine elicits neutralizing antibodies that protect humanized mice against EBV challenge

**DOI:** 10.1038/s41541-024-00907-y

**Published:** 2024-06-26

**Authors:** Kristina R. Edwards, Harman Malhi, Karina Schmidt, Amelia R. Davis, Leah J. Homad, Nikole L. Warner, Crystal B. Chhan, Samuel C. Scharffenberger, Karen Gaffney, Troy Hinkley, Nicole B. Potchen, Jing Yang Wang, Jason Price, M. Juliana McElrath, James Olson, Neil P. King, Jennifer M. Lund, Zoe Moodie, Jesse H. Erasmus, Andrew T. McGuire

**Affiliations:** 1 Vaccine and Infectious Disease Division, Fred Hutchinson Cancer Center, Seattle, WA USA; 2 Department of Global Health, University of Washington, Seattle, WA USA; 3 HDT Bio, Seattle, WA USA; 4 Department of Laboratory Medicine and Pathology, University of Washington, Seattle, WA USA; 5 Department of Biochemistry, University of Washington, Seattle, WA USA; 6 Institute for Protein Design, University of Washington, Seattle, WA USA; 7 Ben Towne Center for Childhood Cancer Research, Seattle Children’s Research Institute, Seattle, WA USA; 8 Department of Medicine, University of Washington, Seattle, WA USA

**Keywords:** RNA vaccines, Viral infection

## Abstract

Epstein-Barr virus (EBV) is associated with several malignancies, neurodegenerative disorders and is the causative agent of infectious mononucleosis. A vaccine that prevents EBV-driven morbidity and mortality remains an unmet need. EBV is orally transmitted, infecting both B cells and epithelial cells. Several virally encoded proteins are involved in entry. The gH/gL glycoprotein complex is essential for infectivity irrespective of cell type, while gp42 is essential for infection of B cells. gp350 promotes viral attachment by binding to CD21 or CD35 and is the most abundant glycoprotein on the virion. gH/gL, gp42 and gp350, are known targets of neutralizing antibodies and therefore relevant immunogens for vaccine development. Here, we developed and optimized the delivery of several alphavirus-derived replicon RNA (repRNA) vaccine candidates encoding gH/gL, gH/gL/gp42 or gp350 delivered by a cationic nanocarrier termed LION™. The lead candidate, encoding full-length gH/gL, elicited high titers of neutralizing antibodies that persisted for at least 8 months and a vaccine-specific CD8^+^ T cell response. Transfer of vaccine-elicited IgG protected humanized mice from EBV-driven tumor formation and death following high-dose viral challenge. These data demonstrate that LION/repRNA-gH/gL is an ideal candidate vaccine for preventing EBV infection and/or related malignancies in humans.

## Introduction

EBV is a ubiquitous gamma herpesvirus^1^. While primary infection typically is asymptomatic, it can result in infectious mononucleosis^2,3^. EBV was the first virus shown to be oncogenic in humans and is associated with approximately 358,000 new cases of cancer resulting in 209,000 deaths each year^4,5^. Beyond its contribution to the global cancer burden, EBV has been linked to autoimmune conditions such as rheumatoid arthritis and multiple sclerosis^6–10^. A successful vaccine against EBV that can prevent infection and/or reduce disease remains an unmet need that would lessen global morbidity and mortality linked to these conditions.

Several vaccine candidates are in various stages of preclinical and clinical development, most are derived from surface glycoproteins involved in attachment and entry^11^. EBV primarily infects B cells and epithelial cells and has distinct attachment and entry pathways for each^12^. The viral fusion machinery gH, gL, and gB are critical for infection irrespective of cell type^12,13^. gH and gL form a 1:1 heterodimeric complex that acts as a regulator of membrane fusion. Upon binding one or more host cell surface receptors, gH/gL relays a triggering signal to the fusogen gB^13,14^. EBV infection of B cells is initiated by attachment to CD21 or CD35 by the viral protein gp350^15,16^. B cell infection requires an additional viral protein, gp42 that forms a tripartite complex with gH and gL^17^. Binding of gp42 to human leukocyte antigen class II molecules on the B cell surface leads to triggering of gB-mediated fusion through the gH/gL-gp42 complex^18–20^. Virus lacking gp350 shows reduced infectivity^21^, while gp42 is essential for infection^22^.

In naturally infected individuals, gH/gL has been identified as the major target of antibodies that prevent EBV infection of epithelial cells, whereas antibodies that neutralize EBV infection of B cells are directed at gp350 followed by gH/gL and gp42^23^. Multiple neutralizing monoclonal antibodies (mAbs) have been isolated against the gH/gL glycoprotein complex that can block viral entry into both epithelial cells and B cells in vitro^23–26^. The details of the interactions between gH/gL and cell surface receptors, and the molecular mechanisms by which it activates gB, are poorly understood. Consequently, the mechanisms by which neutralizing mAbs against gH/gL prevent membrane fusion are not known. Nevertheless, passive transfer of gH/gL mAbs can protect against viral challenge in animal models of EBV infection^26,27^. Similarly, anti-gp42 mAbs have been isolated from non-human primates (NHPs), mice, and rabbits that neutralize EBV infection of B cells, and some have been shown to prevent experimental EBV infection in animal models^28–33^. Collectively, these findings indicate that gH/gL, gp42 and gp350 are strong candidates for vaccine development. To this end, our group and others have developed protein nanoparticles displaying EBV gH/gL, gH/gL/gp42, or gp350 that elicit high titers of binding and neutralizing antibodies that protect against lethal EBV challenge in mice with humanized immune systems^23,34–36^.

As an alternative to protein-based vaccines, we sought to leverage advances made in nucleic acid-based delivery to deliver EBV glycoprotein immunogens. An attenuated variant of the Venezuelan equine encephalitis virus, TC-83 strain, has been used to generate self-amplifying replicon RNA (repRNA) vaccines where the viral RNA replication complex is intact, but the structural genes are replaced with a gene of interest. Delivery of repRNA into cells promotes synthesis of antigen-encoding RNA that self-adjuvants by triggering innate immune responses and promoting antigen cross-priming which enhances humoral and cellular immune responses compared to conventional mRNA. Delivery of repRNAs encoding diverse viral antigens with a cationic nanocarrier (termed LION) has been shown to elicit high titers of antibodies as well as T cell responses in several preclinical animal models^37–41^. Moreover, this platform has led to the development of an effective SARS-CoV-2 vaccine licensed for emergency clinical use^42,43^. LION -formulated repRNA offers significant advantages over other RNA vaccine platforms, including limiting the dissemination of RNA to the injection site which induces antigen-specific adaptive immunity while avoiding systemic inflammation^37^.

Here, we evaluated the ability of several LION/repRNA encoded gH/gL, gH/gL/gp42, and gp350 derivatives to elicit binding and neutralizing antibodies. After optimization of the construct and dosing regimen, polyclonal antibodies were evaluated for their ability to prevent EBV infection in a humanized mouse model. Passive transfer of antibodies elicited by vaccination with LION/repRNA encoding full-length gH/gL prior to challenge with a high dose of EBV provided protection from lethality. LION/repRNA-elicited antibodies also reduced viral load, prevented splenomegaly, and precluded splenic tumor formation. In contrast, mice given IgG elicited by a more conventional gH/gL protein subunit vaccine became viremic, developed splenic tumors, and in some cases required euthanasia. In addition to eliciting superior humoral responses, the repRNA encoded gH/gL elicited a higher frequency of vaccine specific CD8^+^ T cell responses. Collectively, these results demonstrate that LION/repRNA encoded gH/gL is an attractive alternative to recombinant subunit vaccines capable of eliciting high titers of protective antibodies that could be augmented by cellular immune responses.

## Results

### Characterization of repRNA encoded gH/gL and gH/gL/gp42

**Fig. 1:**
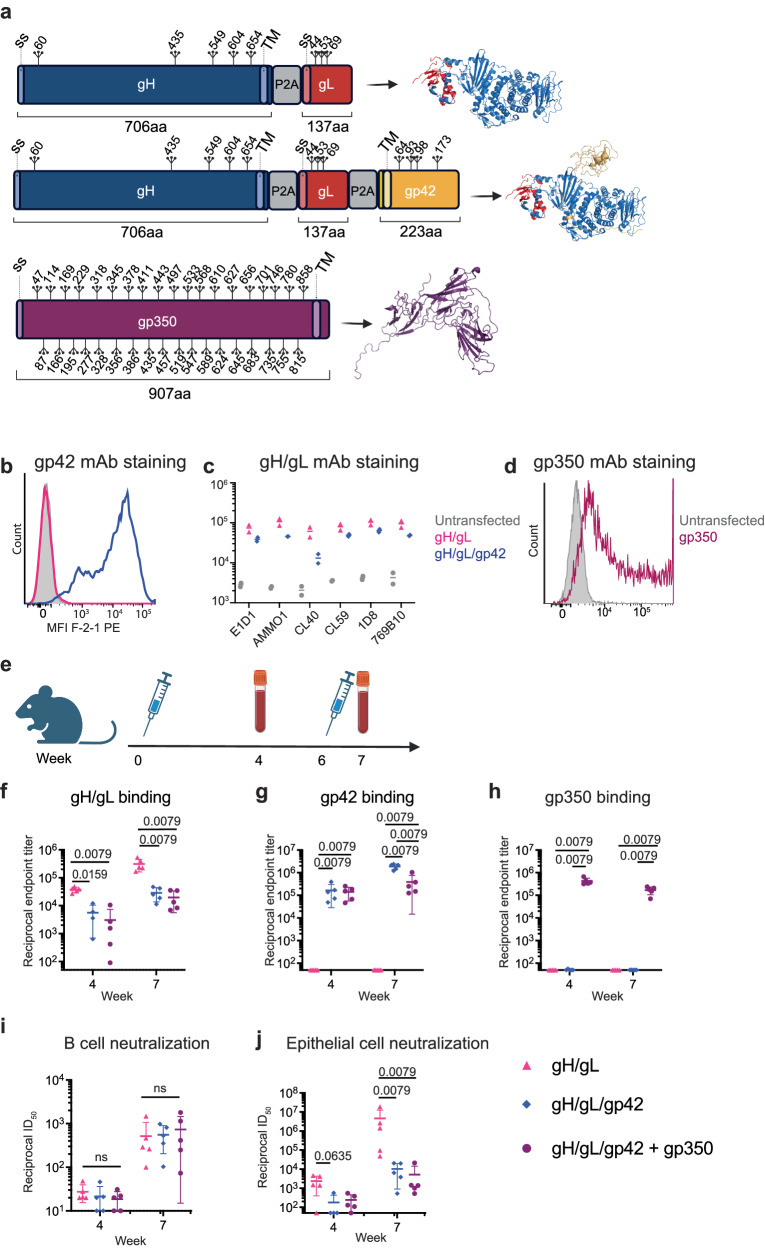
Development and immunogenicity of repRNAs encoding EBV gH/gL, gH/gL/gp42, and gp350. **a** 2D-schematics representing repRNA inserts encoding gH and gL, gH, gL, and gp42, or gp350. For tandem constructs, each glycoprotein is separated by a P2A peptide as indicated. Signal sequences (SS), transmembrane domains (TM), and positions of putative N-linked glycosylation sites are indicated. The expected 3D polypeptides are depicted to the right of each 2D schematic. The 3D gH/gL and gH/gL/gp42 complexes were created using PyMOL based on PDBID 6C5V. The 3D structure of gp350 was created using PyMOL based on PDBID 2H6O. **b** Staining of 293 cells transfected with repRNAs encoding gH/gL and gH/gL/gp42 with the anti-gp42 mAb F-2-1. **c** 293 cells from (**b**) were stained with a panel of anti-gH/gL mAbs as indicated. Each dot represents the mean fluorescence intensity (MFI) of a technical replicate (*n* = 3). The MFI of PE-positive cells is shown for gH/gL and gH/gL/gp42 while the PE-MFI of all cells is shown for the transfected control. **d** Staining of 293 cells transfected with repRNA encoding gp350 with the anti-gp350 mAb 72A1. **e** Immunization and bleed schedule for evaluating the repRNA constructs in (**a**). Reciprocal endpoint binding titers to gH/gL (**f**), gp42 (**g**), or gp350 (**h**) in sera at weeks 4 and 7 measured by ELISA as indicated. The ability of sera from mice immunized with repRNA encoding gH/gL, gH/gL/gp42, or gH/gL/gp42 + gp350 to neutralize EBV infection of B cells (**i**), or epithelial cells (**j**) as indicated. Each dot represents an individual mouse (*n* = 5), the horizontal bars represent the means, and the error bars represent the standard deviation in (**f**–**j**). Statistical differences were determined using a Mann-Whitney Test. **a** and **e** were created using BioRender.com.

To enable co-delivery of both gH and gL or gH, gL, and gp42 on a single repRNA, we designed two constructs. The first encodes gL and then full-length gH (including the transmembrane and cytoplasmic domains), while the second includes gH, gL, and gp42. The viral genes are delivered in tandem separated by ribosomal skipping P2A peptides and an upstream furin cleavage site to facilitate the removal of residual P2A residues on the N-terminal protein following translation (Fig. 1a). This tandem expression strategy ensures that the vaccine polypeptides are produced in each cell and promotes proper co-folding of gH/gL and/or gH/gL/gp42. We also developed a repRNA encoding full-length gp350 (Fig. 1a). mRNA from each repRNA construct was transcribed and capped in vitro via T7 polymerase and vaccinia capping enzymes, respectively.

repRNAs were transfected into 293 cells and stained with the anti-gp42 mAb F-2-1^30^. Cells transfected with repRNA encoding gH/gL/gp42, but not cells transfected with repRNA encoding gH/gL, stained positive for F-2-1, verifying expression of gp42 (Fig. 1b). The same cells were stained with a panel of fluorescently labeled neutralizing mAbs against gH/gL (Fig. 1c). Anti-gH/gL mAb-positive cells were observed in cell pools transfected with both constructs, indicating that the gH/gL is expressed and presents relevant neutralizing epitopes. In all cases, the intensity of mAb staining among positive cells was lower for repRNA encoding gH/gL/gp42 as compared to gH/gL alone. Staining with the CL40 mAb was substantially lower in cells transfected with gH/gL/gp42, consistent with steric occlusion of the CL40 epitope by gp42 which inhibits binding of this mAb^44^. Cells transfected with repRNA encoding gp350 were readily stained with the anti-gp350 72A1 mAb^45^ (Fig. 1d).

To evaluate the immunogenicity of these constructs, 10 µg of gH/gL or gH/gL/gp42 repRNA was formulated with LION and delivered to groups of 5 C57BL/6 mice at weeks 0 and 6. Another group received 10 µg of gH/gL/gp42 repRNA/LION co-injected with 10 µg of gp350 repRNA/LION at weeks 0 and 6. Blood was collected 4 weeks after the first immunization and 1 week after the second immunization for serological analyses (Fig. 1e). All constructs were immunogenic and elicited antigen-specific binding antibodies in sera (Fig. 1f–h). Delivery of repRNA gH/gL alone elicited higher gH/gL binding titers than repRNA gH/gL/gp42 (Fig. 1f). To monitor neutralization in epithelial cells, we used the SVKCR2 cell line that stably expresses CD21, which promotes cellular attachment of virions via gp350, improving the otherwise poor infectivity of epithelial cells in vitro^46^. Raji cells were used to measure EBV neutralizing titers against B cell infection. Both the gH/gL and gH/gL/gp42 repRNAs elicited neutralizing titers against EBV infection of epithelial and B cells that were boosted by a second immunization (Fig. 1i,j). There were no differences in the ability of the two constructs to elicit antibodies that neutralize EBV infection of B cells (Fig. 1i), and both elicited titers that were higher than those in the serum of EBV seropositive donors (Supplementary Fig. 1). Similarly, both the gH/gL and gH/gL/gp42 repRNAs elicited neutralizing titers against EBV infection of epithelial cells that were higher than those measured in serum of EBV carriers (Supplementary Fig. 1), but the epithelial cell neutralizing titers elicited by repRNA encoding gH/gL/gp42 were significantly lower than those elicited by repRNA encoding gH/gL (Fig. 1j). Despite eliciting high gp350 binding titers (Fig. 1h), the co-delivery of the gp350 repRNA with gH/gL/gp42 repRNA did not improve the neutralizing titers against EBV infection of B cells or epithelial cells compared with gH/gL/gp42 alone (Fig 1i, j).

### Dose optimization for LION/repRNA gH/gL vaccination

**Fig. 2:**
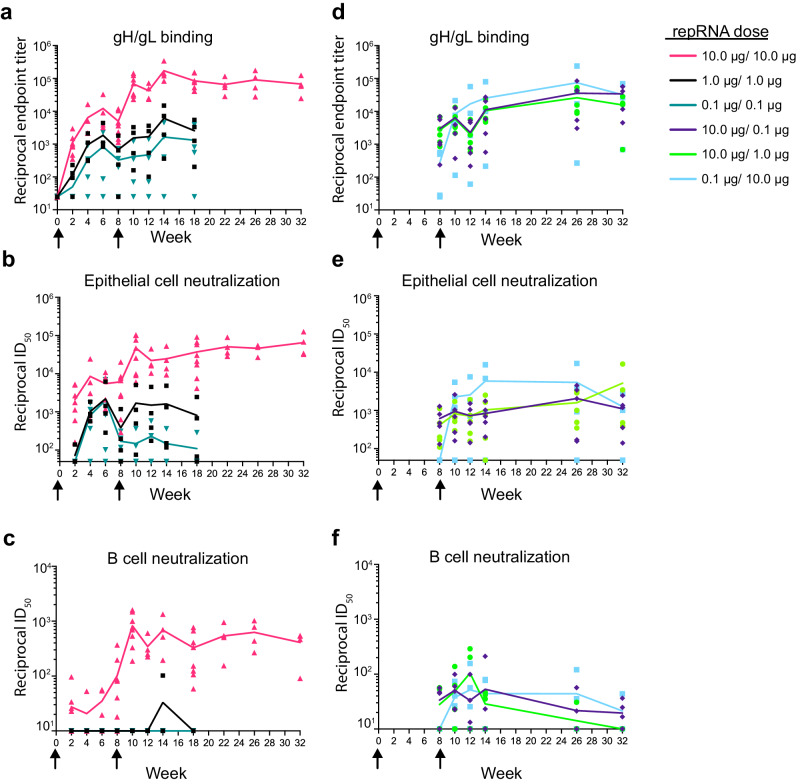
Dose optimization of gH/gL LION/repRNA. **a** Kinetics of reciprocal gH/gL endpoint binding titers. Half-maximal neutralizing titers against EBV infection of epithelial (**b**) or B cells (**c**) following equal doses of LION/repRNA encoded gH/gL delivered at weeks 0 and 8 as indicated. The gH/gL binding (**d**) and EBV neutralizing titers measured in epithelial (**e**) and B cell (**f**) infection assays following two unequal doses of repRNA encoded gH/gL delivered at weeks 0 and 8 as indicated. Each data point represents the average of two technical replicates for an individual mouse at each timepoint (*n* = 4 per group for equal 0.1 and 1 µg prime/boost doses, *n* = 8 for equal 10 µg prime/boost doses, and *n* = 5 per group for mixed dose prime/boost) and lines connect the means. Arrows indicate the time of immunization. See Table S1 for statistical comparisons.

Given that gH/gL elicited higher neutralizing titers against EBV infection of epithelial cells than gH/gL/gp42 and gH/gL/gp42 + gp350 did when delivered at weeks 0 and 6 in our pilot experiments (Fig. 1j), we sought to optimize the dosing schedule of repRNA encoded gH/gL. We first compared the immunogenicity of repRNA where the order of gH and gL were swapped on a tandem expression construct. We observed slightly higher anti-gH/gL titers when gL preceded gH (Supplementary Figure 2), so we carried out subsequent immunogenicity experiments using this construct. Groups of 4 C57BL/6 mice received 0.1 μg, 1 μg, and 10 μg of LION/repRNA gH/gL at week 0. The animals were bled biweekly and the gH/gL-binding endpoint titers were monitored in near-real time. Following the priming immunization, we observed a dose-dependent increase in the binding titers until week 6, followed by a slight waning by week 8 (Fig. 2a and Supplementary Table 1), at which point we opted to deliver a second immunization with the same dose. The endpoint binding titers were boosted in the animals that received a second 10 μg dose. A slight boost was also observed in the animals that received a 1 μg dose, but not in the animals that received a 0.1 μg second dose (Fig. 2a).

We examined the ability of the immune sera to neutralize EBV infection of epithelial and B cells in vitro. The trends observed in the endpoint binding titer kinetics were mirrored in the neutralizing titers against epithelial cell infection. Following the prime, there was a dose-dependent increase in epithelial cell neutralizing titers until week 6 that waned slightly by week 8 and was boosted by a second dose in the 10 μg and 1 μg, but not the 0.1 μg, groups (Fig. 2b).

Only the 10 μg dose elicited antibodies capable of neutralizing EBV infection of B cells after a single immunization (Fig. 2c). Two weeks after a second immunization with a 10 μg dose, these titers were boosted over 10-fold to a reciprocal half-maximal inhibitory dilution (ID_50_) near 1 × 10^3^. Weak transient neutralizing titers were observed 6 weeks after a second dose with 1 μg of LION/repRNA gH/gL, while the 0.1 μg dose failed to elicit B-cell neutralizing antibodies at any timepoint (Fig. 2c).

In our initial experiments, all animals were euthanized at week 18. To measure durability of the antibody responses, a second cohort of animals (*n* = 4) received 10 μg of LION/repRNA at weeks 0 and 8 and the antibody responses were monitored for a total of 32 weeks. We observed that the binding and neutralizing titers were maintained during this time (Fig. 2a–c).

Varying the dose of the prime and boost can impact vaccine responses. For example, administration of a fractional dose boost in a malaria vaccine trial elicited superior antibody responses compared to a higher dose boost^47^. Conversely, a low dose prime followed by a higher dose boost regimen of a SARS-CoV-2 vaccine showed greater immunogenicity and efficacy than a high dose prime/boost regimen^48,49^. To explore whether varying the prime and boost dose affects the immunogenicity of LION/repRNA gH/gL, we evaluated two de-escalating dose regimens, 10 μg/1 μg and 10 μg/0.1 μg, and one escalating dose 0.1 μg/10 μg regimen. Animals that received a 10 μg prime had binding and B cell neutralizing titers comparable to the original cohort of mice from the 10 µg/10 µg group at week 8 (Fig. 2a, d, c, f), while the epithelial cell neutralizing titers were lower in the mice in the 10 μg/1 μg and 10 μg/0.1 μg at week 8 (Fig. 2b,e and Supplementary Table 1). None of the binding or neutralizing titers were boosted when the second dose was lower (Fig. 2d, e, f, green and dark blue curves). After the initial dose, the 0.1 μg/10 μg group had low gH/gL binding but no epithelial cell neutralizing titers (Fig. 2d, e, light blue curve), or B cell neutralizing titers by week 8 (Fig. 2f, light blue curve). However, after the boost immunization of 10 μg at week 8, there were slightly higher gH/gL binding titers and epithelial cell neutralizing titers than the other mixed dose groups (Fig. 2d, e, light blue curve), but similar B cell neutralizing titers (Fig. 2f light blue curve and Supplementary Table 1). Although there were differences in the number of mice used in each group (*n* = 4 – *n* = 8) and up to ~10-fold differences in the binding and neutralizing titers among individual mice within each group (Fig. 2a–f), across all prime/boost regimens evaluated, a 10 μg prime followed by a 10 μg boost elicited binding and neutralizing titers that were significantly higher than those elicited in the other groups at most timepoints following the second immunization (Supplementary Table 1).

### repRNA encoding membrane-anchored gH/gL monomer is more immunogenic than the gH/gL ectodomain

**Fig. 3:**
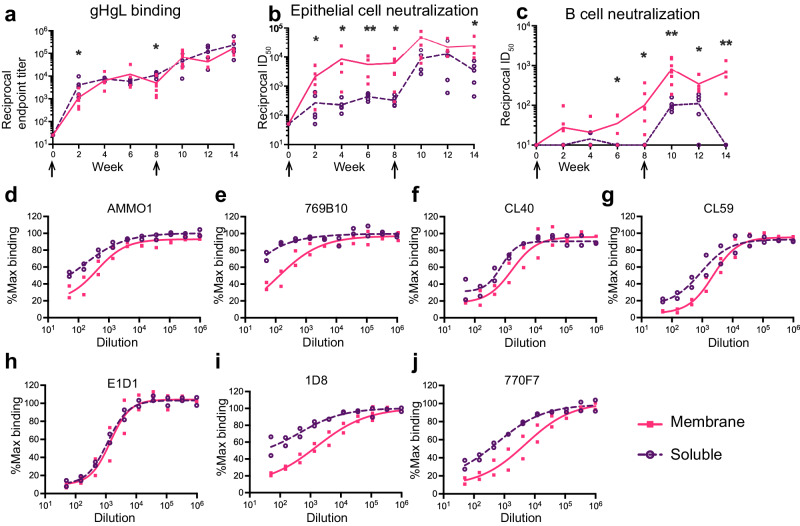
Full-length gH/gL elicits higher neutralizing titers than the gH/gL ectodomain when delivered by LION/repRNA. **a** Reciprocal gH/gL endpoint binding titers elicited by LION/repRNA encoding the soluble gH/gL ectodomain measured by ELISA as indicated. Titers elicited by the 10 μg prime-boost regimen with full-length membrane-anchored gH/gL from Fig. 2 are shown for comparison. The sera from (**a**) was evaluated in neutralization assays carried out in epithelial (**b**), or B cells (**c**). Each dot represents the average of three technical replicates for an individual mouse at each timepoint (*n* = 8 for full-length gH/gL and *n* = 5 for gH/gL ectodomain) and the lines connect the means. The arrows indicate the time of immunization. Asterisks denote a statistically significant difference between the two groups at a given time point determined using a Mann-Whitney test where * indicates *p* ≤ 0.05 and ** indicates *p* ≤ 0.01. **d**–**j** Pooled immune sera from mice vaccinated with LION/repRNA encoding full-length and gH/gL ectodomain were evaluated for their ability to compete for binding to EBV gH/gL with the indicated mAbs by competitive ELISA. Each dot represents a technical replicate with a line connecting the mean.

After determining an optimal dose regimen for LION/repRNA gH/gL vaccination, we next compared the immunogenicity of a secreted gH/gL ectodomain with full-length gH/gL. To produce the soluble ectodomain, gH was truncated at amino acid 679 in the gL-P2A-gH construct (Supplementary Fig. 2a). 10 μg of LION/repRNA encoding the ectodomain was delivered at weeks 0 and 8, and sera samples were collected through week 14. After the first immunization, the ectodomain elicited gH/gL binding titers that were nearly identical to full-length gH/gL (Fig. 3a), however the epithelial cell neutralizing antibody titers were ~10-fold lower than full length gH/gL, and B cell neutralizing titers were not elicited by week 8 (Fig. 3b, c). A second immunization with the ectodomain boosted the epithelial cell neutralizing titers to similar levels achieved with full-length gH/gL, but the B cell neutralizing titers were ~5-fold lower and the neutralizing titers decayed more rapidly in both assays (Fig. 3a–c). We sought to discern whether the observed differences in neutralizing activity were due to differential epitope recognition between the serum antibodies elicited by these two constructs. Week 12 sera from both groups was pooled and evaluated for its ability to compete with the binding of mAbs with defined epitopes on gH/gL, including AMMO1^25^, 769B10^23^, E1D1^50^, CL40^31^, CL59^31^, 1D8^26^, and 770F7^24^. With the exception of E1D1 which binds an epitope entirely on gL^51^, sera elicited by full-length gH/gL competed the binding of all mAbs more potently than sera elicited by the gH/gL ectodomain (Fig. 3d–j). Collectively, these data demonstrate that full-length LION/repRNA gH/gL elicits a qualitatively different antibody response than the LION/repRNA gH/gL ectodomain, resulting in higher neutralizing titers.

### Membrane-retained gH/gL monomer repRNA immunization is more immunogenic than secreted gH/gL multimers

**Fig. 4:**
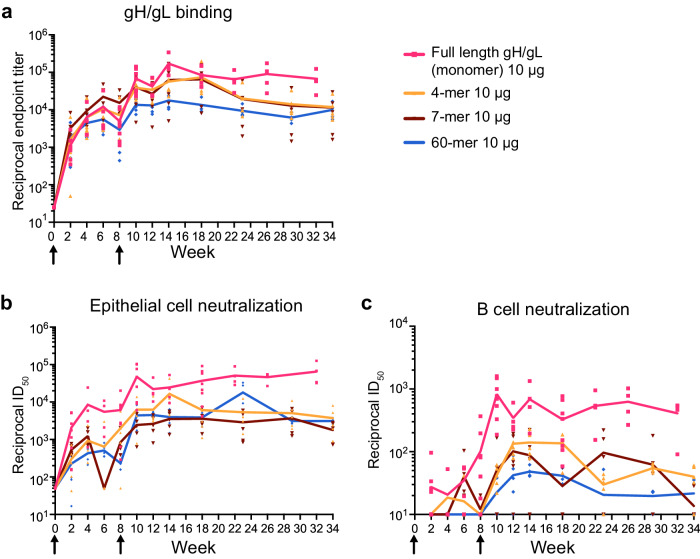
Full-length gH/gL elicits higher neutralizing titers than multimeric gH/gL ectodomain constructs when delivered by LION/repRNA. **a** Reciprocal endpoint gH/gL binding titers from mice immunized with LION/repRNA encoding gH/gL 4-mer, gH/gL 7-mer and gH/gL 60-mer were measured by ELISA. Titers from two immunizations with 10 μg full-length gH/gL (from Fig. 2) are included for comparison as indicated. EBV neutralizing titers in the serum from the mice in (**a**) were measured in epithelial (**b**) and B cell (**c**) infection assays. Each dot represents the average of three technical replicates for an individual mouse at each timepoint (*n* = 5 per group in multimer construct vaccinations, *n* = 8 for full-length gH/gL) and the lines connect the means. The arrows indicate the time of immunization. See Table S2 for statistical comparisons.

We and others have previously shown that multimerization of gH/gL through genetic fusion to self-assembling nanoparticles substantially improves its immunogenicity when delivered as recombinant protein^23,34,35^. Therefore, we evaluated LION/repRNA delivery of multimeric gH/gL. Mice were immunized with 10 μg of LION/repRNA encoding the gH/gL ectodomain presented as different multimeric constructs, a 4-mer, 7-mer, and a 60-mer that we previously developed as protein subunit vaccines^34^. After the first immunization and through week 8, similar gH/gL binding titers were elicited by all constructs (Fig. 4a and Supplementary Table 2). After the second immunization, the binding titers elicited by the membrane-anchored monomer were comparable to those elicited by the 4-mer and 7-mer, but higher than those elicited by the gH/gL 60-mer (Fig. 4a and Supplementary Table 2). The differences in the neutralizing titers between the groups that received the membrane-anchored gH/gL and multimeric constructs were starker. The membrane-anchored monomer elicited higher titers than the multimeric constructs in the B cell and epithelial cell neutralization assays at nearly every timepoint tested from weeks 8–34 (Fig. 4b, c and Supplementary Table 2).

### Passive transfer of IgG elicited by LION/repRNA gH/gL protects humanized mice from lethal EBV challenge

Having established that two 10 μg doses of LION/repRNA-encoded full-length gH/gL delivered at weeks 0 and 8 showed favorable immunogenicity, we evaluated whether antibodies elicited by this regimen are protective in vivo. To do this, we undertook a passive transfer and challenge experiment in humanized mice. The use of humanized mice as a small animal model of EBV infection is well established^52–57^. In short, highly immunocompromised NOD *scid* gamma mice are irradiated and engrafted with human CD34^+^ cells, which then reconstitute the human hematopoietic compartment in the mouse. This allows for EBV infection of human B cells in vivo. Humanized mice generate poor antibody responses^58^, therefore it was necessary to passively transfer IgG from immunized C57BL/6 mice prior to EBV challenge. This approach has been previously used to evaluate the efficacy of anti-EBV mAbs and vaccine-elicited antibodies^24,26,27,34,35^.

**Fig. 5:**
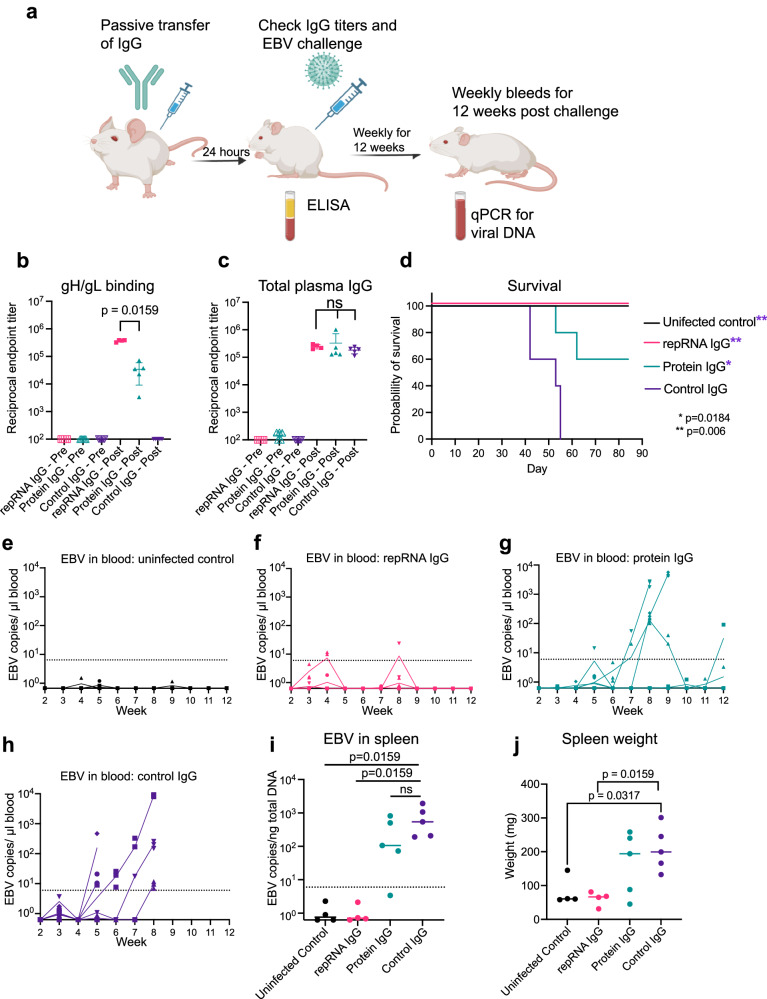
IgG elicited by LION/repRNA encoding gH/gL protects humanized mice from lethal EBV challenge. **a** Humanized mice received IgG via intraperitoneal injection that was harvested from one of three groups: mice immunized with gH/gL protein, mice immunized with LION/repRNA encoding gH/gL, or unimmunized control mice. 24 h later, mice were bled and challenged with EBV, bled weekly starting 2 weeks post-challenge, and then euthanized at week 12 or earlier if humane endpoints were met. Created using BioRender.com. Reciprocal endpoint titers of gH/gL binding (**b**) and total IgG (**c**) were measured at the time of challenge. Each dot represents an individual mouse (*n* = 4 repRNA IgG, *n* = 5 Protein IgG and Control IgG), the horizontal bars represent the means, and the error bars represent the standard deviation in (**b**) and (**c**). **d** Survival of mice after challenge. Significant differences between each group and the IgG control were determined using a log-rank Mantel-Cox test. Viral DNA in the peripheral blood of control mice (**e**) and mice that received passive transfer of repRNA/LION gH/gL elicited IgG (**f**) recombinant gH/gL elicited IgG (**g**) or control IgG (**h**). Three technical replicates were run. **i** Viral DNA was quantified in splenic DNA extracts at necropsy. Each dot represents the average of three technical replicates for an individual mouse, the bar represents the median copy number per group, and the dashed line indicates the limit of detection. **j** Spleen weights at necropsy, each dot represents an individual mouse, and bar represents the median weight. Significant differences between all pairs of groups were assessed using Mann-Whitney tests in (**i**) and (**j**).

Twenty-five C57BL/6 mice were immunized with 10 μg of LION/repRNA encoding full-length monomeric gH/gL at weeks 0 and 8. To compare this to a more conventional recombinant vaccine, another group of 25 mice were given two doses of 5 μg of purified monomeric gH/gL ectodomain protein formulated with Sigma Adjuvant System at weeks 0 and 8 (Fig. 5a). At week 12, mice were euthanized, and IgG was harvested from pooled sera. IgG purified from mice immunized with repRNA showed stronger binding to gH/gL than IgG purified from protein-vaccinated mice (Supplementary Fig. 3).

After verifying the successful engraftment of human CD45^+^ cells and development of CD19^+^ B cells in humanized mice (Supplementary Fig. 4), 500 μg of purified IgG from mice immunized with gH/gL encoded by LION/repRNA or protein was delivered to groups of 4 humanized mice. An additional five humanized mice received 500 μg of IgG purified from unimmunized C57BL/6 mice. The next day, mice were bled to confirm IgG transfer and challenged via intravenous injection with 33,000 Raji infectious units of EBV. Five mice that did not receive IgG transfer remained unchallenged and served as an uninfected control group (Fig. 5a). No animals had serum IgG prior to transfer, but all had similar levels at the time of challenge, confirming transfer of equal amounts of IgG in all study animals (Fig. 5c). The LION/repRNA group had significantly higher anti-gH/gL ELISA titers compared to the protein group (Fig. 5b) consistent with the higher activity of the purified IgG (Supplementary Fig. 3). The gH/gL binding titers in humanized mice in the repRNA IgG group at the challenge were comparable, on the order of 1 × 10^5^, to those elicited by 2 doses of 10 μg of LION/repRNA in C57BL/6 mice (compare Fig. 2a and Fig. 5b).

Starting 2 weeks post challenge and continuing weekly for 10 weeks, mice were weighed three times a week (Supplementary Fig. 5) and bled weekly. To monitor for infection, DNA was extracted from whole blood and qPCR was used to measure viral DNA (Fig. 5e–h). At week 12, or sooner if humane endpoints were met, mice were euthanized and spleens were examined for splenomegaly, tumors, and the presence of viral DNA (Fig. 5i, j and Supplementary Fig. 6).

Following challenge, 100% of the mice in the uninfected control group survived (Fig. 5d) and lacked detectable viral DNA in the blood (Fig. 5e) and the spleen (Fig. 5i). In contrast, none of the mice in the group that received control IgG survived beyond 8 weeks (Fig. 5d), all were viremic (Fig. 5h) and had high levels of viral DNA in the spleen (Fig. 5i). These mice also developed splenomegaly (Fig. 5j) and had splenic tumors (Supplementary Fig. 6). Three of the mice in the protein group developed viremia by week 8 (Fig. 5g) and two required euthanasia at weeks 8 and 9 (Fig. 5d), while the other three survived until week 12 (60% survival). Four of five mice in the protein group had elevated levels of viral DNA in the spleen (Fig. 5i), three developed splenomegaly (Fig. 5j), and two developed splenic tumors (Supplementary Fig. 6). We note that one mouse in the protein IgG group had notably lower gH/gL binding activity than the others despite equal transfer of total IgG (Fig. 5b, c and Supplementary Table 3, mouse 840). This mouse also had the highest peak viremia, largest spleen, and highest splenic viral DNA load (Fig. 5g, i and j and Supplementary Table 3). In the repRNA group, only one mouse exhibited transient low-level viremia (Fig. 5f) and 100% of the mice survived for 12 weeks following challenge (Fig. 5d). At week 12, the spleen weights were comparable to the uninfected controls (Fig. 5j) and free of viral DNA (Fig. 5i) and tumors (Supplementary Figure 6). In sum, immunization with gH/gL repRNA elicited higher gH/gL IgG titers that provided superior protection from lethal EBV challenge, compared with a conventional protein-based vaccine formulation in a humanized mouse model.

### LION/repRNA gH/gL elicits higher cellular responses than immunization with adjuvanted recombinant gH/gL

**Fig. 6:**
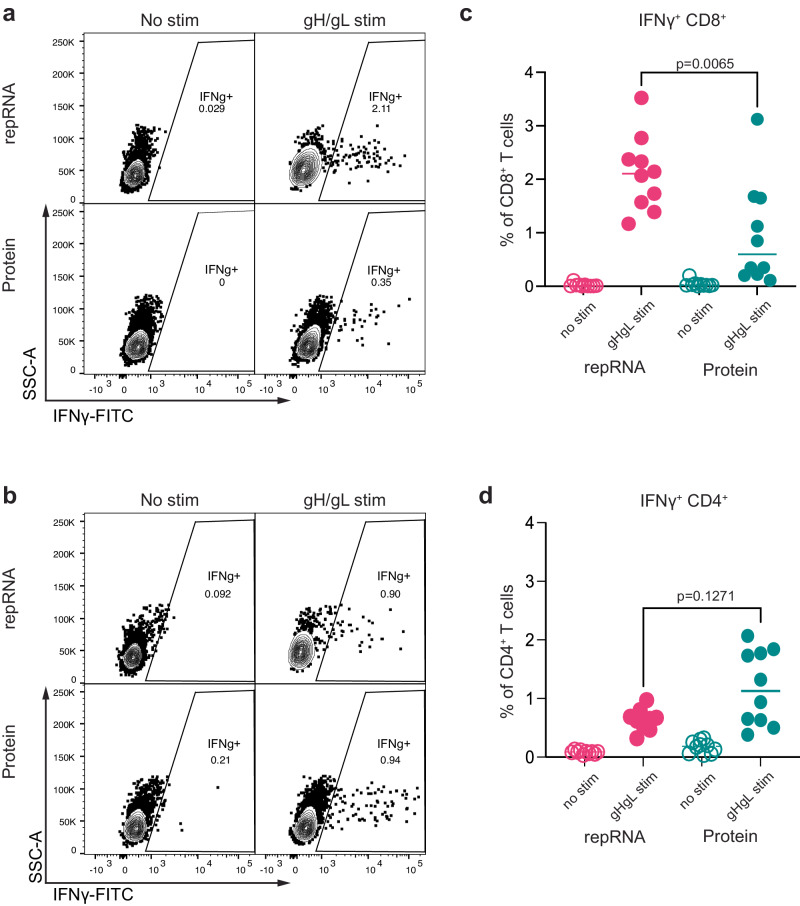
LION/repRNA gH/gL elicits stronger vaccine-specific CD8^+^ T cell responses than the recombinant gH/gL ectodomain. Exemplary IFNɣ^+^ CD8^+^ (**a**) and IFNɣ^+^ CD4^+^ (**b**) T cell responses in unstimulated or gH/gL-stimulated splenocytes from mice immunized with recombinant gH/gL or LION/repRNA gH/gL using an intercellular staining assay as indicated. See Fig. S7 for gating strategy. Frequency of IFNɣ^+^ CD8^+^ T (**c**) and CD4^+^ T (**d**) cells in unstimulated and gH/gL-stimulated splenocytes from mice immunized with recombinant gH/gL or LION/repRNA gH/gL as indicated. Each dot represents an individual mouse (*n* = 10 per group), The bars represent the mean. Significant differences were determined using a Mann-Whitney test in (**c**) and (**d**).

The challenge experiments demonstrated that antibodies elicited by repRNA immunization conferred superior antibody-mediated protection compared with antibodies elicited by protein. To compare cellular immunity elicited by both vaccines, we collected splenocytes from twenty animals used to generate IgG for transfer experiments, stimulated them with the recombinant gH/gL ectodomain ex vivo, and analyzed CD4^+^ and CD8^+^ T cells for production of IFNɣ (Fig. 6 and Supplementary Fig. 7). IFNɣ^+^ CD4^+^ and CD8^+^ cells were observed in gH/gL stimulated, but not unstimulated splenocytes from both groups of immunized mice (Fig. 6a–d). In contrast, stimulation with gH/gL did not induce IFNɣ production in CD4^+^ or CD8^+^ splenocytes from unimmunized mice (Supplementary Fig. 7b, c). Mice vaccinated with repRNA had higher frequencies of vaccine-elicited CD8^+^ T cells, as defined by the frequency of IFNɣ^+^ CD8^+^ T cells after splenocyte exposure to gH/gL, than mice immunized with protein did (Fig. 6c). No significant difference in IFNɣ^+^ CD4^+^ T cell vaccine responses was observed (Fig. 6d).

## Discussion

EBV is an important human oncovirus for which there is no vaccine. High titers of neutralizing antibodies against the gH/gL glycoprotein complex protect against experimental EBV infection in animal models, suggesting that eliciting these responses will be an important goal of an effective EBV vaccine. Here we report the development and optimization of a LION/repRNA vaccine encoding the EBV gH/gL glycoprotein complex. Optimization included testing the inclusion of gp42 alone or with gp350, the effect of the order of the two glycoproteins gH and gL in tandem expression constructs, the timing and dosing of repRNA delivery, the effect of soluble versus membrane-anchored antigen, and the delivery of gH/gL fused to different multimeric constructs encoded by repRNA. Favorable immunogenicity of repRNA encoded gH/gL was achieved when the glycoprotein complex was membrane anchored, and the highest dose of repRNA was delivered 8 weeks apart in a prime/boost regimen. This resulted in high titers of binding and neutralizing antibodies that were maintained for up to 32 weeks following immunization.

gp42 is essential for EBV infection and a known target of antibodies that neutralize EBV infection of B cells^23,28–31^. A previous study comparing the immunogenicity of gH/gL with that of gH/gL/gp42 administered as monomeric heterotrimers or ferritin-based nanoparticles in mice found that the inclusion of gp42 elicited slightly higher serum neutralizing titers against EBV infection of B cells and equivalent titers against epithelial cell infection^23^. Here we observed the opposite: equivalent neutralizing titers against B cell infection, but reduced titers against epithelial cell infection when gp42 was co-delivered with gH/gL on the same repRNA. These observations highlight differences between the immunogenicity of multivalent recombinant protein nanoparticles where immunogens are expressed, purified, and characterized ex vivo prior to delivery of a fixed antigen dose – and repRNA, which enables streamlined manufacturing, but the final antigen dose depends on in vivo expression. The reduced binding titers to gH/gL and lower epithelial cell neutralizing titers observed with gH/gL/gp42 repRNA could be caused by lower levels of in vivo expression of gH/gL due to the expression cost of including gp42. This notion is supported by the lower intensity of gH/gL mAb staining to 293 cells expressing gH/gL/gp42 compared to gH/gL and lower binding titers to gH/gL. In addition, the inclusion of gp42 may have shielded the immune system from neutralizing epitopes on gH/gL that preferentially neutralize EBV infection of epithelial cells such as CL40. Indeed, we observe weaker binding of this mAb to 293-expressed gH/gL/gp42 compared with gH/gL alone. Differences in immunogenicity of the two repRNA constructs could also be explained by immune competition that results in lower titers to gH/gL arising from an immunodominance to gp42.

In line with other studies evaluating the immunogenicity of protein nanoparticles in mice, co-delivery of gp350 along with gH/gL/gp42 did not improve the neutralizing titers against EBV infection of epithelial cells or B cells^23,36^. Although the reasons that the repRNA-encoded gH/gL/gp42 elicited lower titers of antibodies capable of neutralizing EBV infection of epithelial cells are unclear and likely multi-factorial, the observation that the inclusion of gp42 and gp350 did not improve the neutralizing potency against EBV infection of B cells motivated further development of repRNA encoding gH/gL alone in our study. Future studies that examine whether the inclusion of gB and/or BMRF2, which are also targets of neutralizing antibodies^25,59–62^, can further improve serum neutralizing activity are warranted.

LION/repRNA delivery of either the full-length membrane-anchored gH/gL, or the soluble gH/gL ectodomain elicited similar gH/gL binding titers. However, the soluble version elicited less potent neutralizing titers against EBV infection of both B cells and epithelial cells, demonstrating a qualitative difference in antibody response to the two antigens. This was further supported by the observation that sera from mice immunized with full-length gH/gL more readily competed for the binding of previously described neutralizing mAbs. These differences in the antibody responses could be due to several non-exclusive possibilities. Membrane anchoring may restrict access to immunodominant, non-neutralizing epitopes that are more exposed to the immune system on the secreted ectodomain. Similarly, membrane anchoring may lead to epitope exposure in a repetitive array that is optimal for engagement of B cell receptors targeting neutralizing epitopes. Although the precise mechanisms that underlie the observed differences between the neutralizing titers elicited by membrane-anchored gH/gL and the ectodomain are not clear, we note that a similar phenomenon was observed when comparing mRNA delivery of membrane-anchored and secreted MERS Spike protein^63^, and that mRNA-based SARS-CoV-2 vaccines are based on full length rather than secreted spike proteins^63–66^. Collectively these observations underscore the importance of evaluating membrane anchoring in nucleic acid delivery of viral glycoprotein vaccine antigens.

We and others previously demonstrated that the immunogenicity of recombinant gH/gL was substantially enhanced by multimeric display on self-assembling nanoparticles^23,34–36^. When delivered by repRNA/LION, gH/gL 4-mers, 7-mers, and 60-mers were less immunogenic than membrane-bound monomeric gH/gL. The reduced antigenicity is likely related to low levels of expression since the yields of these multimeric antigens were inversely correlated with valency when expressed from plasmid DNA in vitro^34^. Consistent with this observation, the binding and neutralizing titers were the lowest in mice immunized with the highest valency repRNA-encoded gH/gL nanoparticle. As noted above, expression of membrane-anchored monomeric gH/gL antigens on the surface of the cell most likely resulted in the display of gH/gL as an array on the cell-surface, which effectively achieved a multimerization effect without compromising the level of gH/gL expression.

Evaluating the efficacy of EBV vaccines in vivo is difficult since the virus has near-obligate tropism for humans, the only natural hosts. NSG mice engrafted with CD34^+^ hematopoietic progenitor cells have been used as small animal models to evaluate the ability of monoclonal^24,26–28,60^ or vaccine elicited antibodies^34–36,59^ to protect against EBV challenge. Here we showed that IgG elicited by repRNA-delivered gH/gL was able to prevent lethality from high-dose EBV challenge, reduce viral load in the blood and spleen, and prevent splenic tumors and splenomegaly. This protection was superior to that achieved by passive transfer of IgG elicited by immunization with a more conventional recombinant gH/gL protein delivered with adjuvant. We previously showed that two immunizations with recombinant multimeric gH/gL nanoparticles elicited similar levels of neutralizing antibodies to those elicited repRNA encoded (monomeric) gH/gL and that passive transfer of the same amount of gH/gL nanoparticle-elicited IgG could also achieve a comparable level of protection in humanized mice. These results demonstrate that repRNA delivery of gH/gL is a viable strategy to elicit high titers of neutralizing antibodies necessary for protection in humanized mice that obviates the challenges of expressing and purifying gH/gL nanoparticles.

The immune incompetence of huCD34 engrafted NSG mice limits the utility of direct immunization, however the protection afforded by transfer or repRNA-elicited IgG clearly highlights a key role of antibodies in anti-EBV immunity. Given that CD8^+^ T cells play a critical role in controlling EBV infection in humans^67^, it stands to reason that antibody-mediated protection elicited by repRNA could be augmented by cellular immune responses^68,69^. In this regard, we note that repRNA elicited higher frequencies of IFNɣ producing CD8^+^ T cells in immune-competent B6 mice.

Humanized mice are not a perfect model for EBV infection as only human-origin B cells support infection and the natural route of oral transmission is not possible^52,54^. Humanized mice may therefore underestimate the relative importance of antibodies capable of neutralizing EBV infection of epithelial cells. Moreover, it is not clear how an intravenous dose of virus in humanized mice compares with an inoculum during a natural oral exposure. Oral challenge of rhesus macaques with the EBV ortholog, rhesus lymphocryptovirus (rhLCV), provides an orthogonal challenge model to evaluate immunogenicity of repRNA vaccines, however antigenic disparity between EBV and rhLCV may belie the predictive efficacy of EBV vaccines in the rhesus macaque model^70^. Nevertheless, further studies to establish the immunogenicity and dosing of LION/repRNA encoding gH/gL in additional small animal models and NHPs will support clinical development. Although we only evaluate immunogenicity in mice in this study, the repRNA/LION platform has demonstrated immunogenicity across species, including mice, rabbits, hamsters, ferrets, and NHPs for other viral vaccines. In all cases, vaccines that induced protective antibody responses in mice, also did so in all other species tested^38–40,42,71^.

Passive transfer of total IgG elicited by recombinant monomeric gH/gL with lower anti-gH/gL activity was less protective than IgG elicited by repRNA in humanized mice. A similar phenomenon was observed comparing recombinant monomeric vs multimeric gH/gL vaccines^34^, suggesting that protection correlates with neutralizing titers. In support of this notion, passive transfer of a neutralizing gH/gL mAb was protective against oral challenge with rhLCV, provided that the antibody was present at adequate levels^27^. Collectively these observations indicate that a neutralizing threshold is required for protection against EBV infection. This implies that a vaccine that induces sterilizing immunity will need to elicit durable high-titer antibodies. We note that the antibody titers elicited by repRNA delivery of gH/gL remained stable for at least 8 months following immunization, suggesting that this platform may be capable of conferring long-term immunity to EBV. Extended durability studies that examine titer longevity and underlying antigen-specific B cell responses are warranted.

In sum, we demonstrate that repRNA/LION delivery of gH/gL elicited high titers of neutralizing antibodies that protected against lethal challenge in a humanized mouse infection model. In addition to eliciting durable neutralizing antibodies, repRNA/LION delivery of gH/gL elicited higher levels of vaccine-specific CD8^+^ T cell responses compared to those elicited by a recombinant gH/gL protein. The robust immunogenicity of repRNA-encoded gH/gL, relative ease of manufacturing, and favorable safety and reactogenicity profile of the delivery platform warrant the development of repRNA EBV vaccines for human clinical trials^37^.

## Methods

### Study design

This study sought to evaluate the ability of alphavirus replicon-encoded delivery of EBV glycoproteins to elicit neutralizing antibodies against EBV. We carried out controlled laboratory experiments to measure binding and neutralizing titers and evaluate the ability of vaccine-elicited antibodies to protect against controlled EBV challenge in humanized mice.

All mice used in our studies were housed with free access to food and water with a 12:12 light:dark cycle. The animal facilities are accredited by the Association for Assessment and Accreditation of Laboratory Animal Care. Mice were handled in accordance with the NIH Guide for the Care and Use of Laboratory Animals, and experiments were approved by the Fred Hutch Cancer Center Institutional Animal Care and Use Committee and Institutional Review Boards. Immunizations and retro-orbital bleeds were carried out under anesthesia, which was induced administering isoflurane, set at 1–5% for 1–2 min in an induction chamber with the flow rate of O_2_ set at 1.0 L/min. Animals under anesthesia were then transferred to a nose cone and continued to receive 1–5% isoflurane at an O_2_ set to 1.0 L/min during injections and retro-orbital bleeds. Mice were euthanized by inducing anesthesia by administering isoflurane, set at 1–5% for 1–2 min in an induction chamber with the flow rate of O_2_ set at 1.0 L/min. Animals under anesthesia were then transferred to a nose cone and continued to receive 1–5% isoflurane at an O_2_ set to 1.0 L/min and then exsanguinated by cardiac puncture using a 25 gauge needle followed by cervical dislocation.

Comparative immunogenicity studies were performed in groups of 4 or 5 C57BL/6 mice between 7 and 10 weeks of age.

Mice were purchased from the Jackson Laboratory and randomized into each group and immunized intramuscularly with repRNA formulated with LION. Each dose administered was split and delivered bi-laterally in the hind legs. Initial experiments compared the immunogenicity of a 10 µg dose of repRNA encoded gH/gL and gH/gL/gp42 in groups of 5 mice. Mice were immunized at weeks 0 and 6 and bled at weeks 4 and 7.

Dose escalation studies were carried out in groups of 4 mice with matched doses of 0.1 µg, 1 µg, and 10 µg delivered at weeks 0 and 8 and bleeds were collected every 2 weeks for 18 weeks. An additional 4 mice were immunized with 10 µg delivered at weeks 0 and 8 and bleeds were collected at weeks 0, 2, 8, 10, 18, 22, 26 and 32. Mixed doses comprised of 10 µg /1 µg, 10 µg /0.1 µg, 0.1 µg /10 µg. Delivered at weeks 0 and 8. Bleeds were collected every 2 weeks from weeks 8–14 and then at weeks 26 and 32. A group of 5 mice were immunized repRNA encoded gH/gL ectodomain were immunized at weeks 0 and 8. Bleeds were collected every 2 weeks until week 14. Groups of 5 mice were immunized repRNA encoding gH/gL fused to self-assembling nanoparticles at weeks 0 and 8. Bleeds were collected every 2 weeks until week 18 and at weeks 23, 29 and 34.

Sera collected from all timepoints was used to measure endpoint binding titers to gH/gL and/or gp42 as appropriate and neutralizing activity against EBV infection of B cells and epithelial cells. Assays were run on independent samples collected from individual mice and were analyzed with respect to treatment group, as described below.

To generate IgG for passive transfer experiments, immunizations were performed in groups of 25 C57BL/6 mice (12 or 13 male and female, varied per group) between 7 and 10 weeks of age. After collecting a pre-bleed, mice were immunized at weeks 0 and 8 with 5 μg of gH/gL monomer in PBS with 50% (v/v) Sigma Adjuvant System (SAS) (Sigma Cat. #S6322) for a total volume of 100 μL per immunization, or mice were immunized with 10 µg of repRNA encoding gH/gL formulated with LION via intramuscular injection as above. Blood was collected via cardiac puncture at week 12 and spleens were collected to analyze vaccine-specific T cell responses. IgG was purified from sera and transferred to humanized mice.

Twenty-five six-week-old NSG mice were irradiated (275R of total body irradiation) and received 1 × 10^6^ CD34^+^ huPBSC in 200 µl PBS through i.v. injection. Eight weeks later, successful human cell engraftment was confirmed by the presence of human CD45^+^ cells in peripheral blood by flow cytometry. 10 weeks post-engraftment, 500 µg of experimental or control antibodies were injected per humanized NSG mouse via intraperitoneal injection (i.p.). 24 h later, mice were bled in the left eye to confirm passive transfer of IgG, and received a dose of EBV B95.8/F67 equivalent to 33,000 Raji infectious units as determined by infection of Raji cells via intravenous injection in the right eye. Each group of mice receiving the same IgG preparation and/or EBV were housed separately from each other. Mice were weighed three times weekly. Beginning at two weeks post-infection, peripheral blood samples were collected to measure the presence of EBV DNA in whole blood. Mice were euthanized twelve weeks post-challenge, or when mice lost 20% of their starting weight. Spleens were photographed and weighed, then DNA was extracted from splenocytes utilizing the DNeasy Blood & Tissue Kit (QIAGEN) according to the manufacturer’s instructions for subsequent viral load analysis.

Group sizes used in these studies are comparable to similar studies previously reported^34,36^.

### Human subjects

Serum was collected from seven adults without HIV and seven with HIV who were recruited at the Seattle HIV Vaccine Trials Unit (Seattle, Washington, USA) as part of the study “Establishing Immunologic Assays for Determining HIV-1 Prevention and Control”, also referred to as Seattle Assay Control (SAC) Cohort. All participants signed informed consent, and the Fred Hutchinson Cancer Center (Seattle, Washington, USA) Institutional Review Board approved the SAC protocol (FHIRB0005567) prior to study initiation. Donors were selected randomly and no considerations were made for age or sex.

### Cell lines

All cell lines were incubated at 37 °C in the presence of 5% CO_2_ and were not tested for mycoplasma contamination. 293-T and 293-6E (human female) were maintained in Freestyle 293 media with gentle shaking. Raji cells (human male) were maintained in RPMI + 10% FBS, 2 mM L-glutamine, 100 U/ml penicillin, and 100 µg/ml streptomycin (cRPMI). 293-2089 cells (human female) were grown in cRPMI containing 100 μg/ml hygromycin^72^. AKATA (human female) B cells harboring EBV in which the thymidine kinase gene has been replaced with a neomycin and GFP cassette virus (AKATA-GFP) were grown in cRPMI containing 350 μg/ml G418^31^. SVKCR2 cells (human male) were grown in DMEM containing 10% fetal bovine serum, 2 mM L-glutamine, 100 U/ml penicillin, 100 μg/ml streptomycin, 10 ng/ml cholera toxin and 400 μg/ml G418^46^.

### Plasmids

Codon optimized cDNA encoding EBV gH (GenBank AFY97969.1) and gL (GenBank: AFY97944.1) separated by a furin cleavage site and a porcine teschovirus-1 (P2A) ribosomal skipping peptide in both orientations (gH-furin-P2A-gL or gL-furin-P2A-gH) with a 5′ Kozak consensus sequence were synthesized by Twist Biosciences and cloned into pVEE-rep encoding the 5′ and 3′ untranslated regions and the nonstructural open reading frame of Venezuelan equine encephalitis virus, strain TC-83, between PflFI and Sac II sites^42,73^, creating pVEE-gH-gL and pVEE-gL-gH. pVEE-gH-gL-gp42 was created by synthesizing a gH-furin-P2A-gL-furin-P2A-gp42 (genBank YP_401672.1) insert and cloning it into pVEE-rep. pVEE-gp350 was created by synthesizing a codon-optimized variant of gp350 (GenBank AFY97937.1) into pVEE rep. pVEE-gL-gH-Ecto was created by introducing a stop codon at AA 170 in gH using the QuikChange II Site-directed mutagenesis kit. pVEE-gL-gH-MDT1100, pVEE-gL-gH-C4b, and pVEE-gL-gH-I3 were produced by amplifying the entire pVEE-gL-gH-Ectodomain plasmid using gene-specific primers and Platinum SuperFi II DNA Polymerase according to the manufacturer’s instructions. Separate sets of gene-specific primers with overlapping homology to the 5′ and 3′ ends of the amplified linear pVEE-gL-gH-Ecto fragment were used to amplify the MDT1100, C4b and I3 multimerization domains from pTT3-gH-IMX313, pCVL-UCOE0.7-SFFV-gH-C153T-cTRP(6)ss-IRES-GFP, and pCVL-UCOE0.7-SFFV-gH--C153T-I3-IRES-GFP plasmids, respectively^34^. The linear pVEE-gL-gH-Ecto fragment was fused to each multimerization domain fragment using the In-Fusion® Snap Assembly Master Mix (Takara) according to the manufacturer’s instructions.

The variable regions corresponding to 769B10 ref.^23^, 1D8^26^, and 770F7^24^ heavy and light chains were synthesized by Integrated DNA Technologies and cloned into pTT3-AMMO1-HC and pTT3-AMMO1LC (for lambda) or pTT3-E1D1LC (for Kappa)^25,27^. All plasmids were confirmed by Sanger sequencing.

### repRNA production

Template pVEE DNAs were linearized by enzymatic digestion with NotI followed by phenol-chloroform treatment and ethanol precipitation. Linearized template was transcribed using the MEGAscript T7 Transcription Kit (Invitrogen) followed by capping with New England Biolabs Vaccinia Capping System as previously described^73^. Capped transcripts were then precipitated in lithium chloride and resuspended in nuclease-free water to a final concentration of 1 mg/ml and analyzed by agarose-gel electrophoresis. All RNA was aliquoted and stored at −80 °C.

### Transfection and cell surface staining of repRNAs in 293 cells

repRNAs were transfected into 30 ml of 293 F cells at a density of 10^6^ cells/ml in Freestyle 293 media (Thermo Fisher Cat. #12338026) using the 293Free transfection reagent (EMD Millipore Cat. #72181-4) according to the manufacturer’s instructions. 24 h later, cells expressing gH/gL, gH/gL/gp42, gp350 or mock-transfected cells were pelleted by centrifugation at 500 × *g* for 5 min and resuspended at 10^6^ cells/ml in PBS containing 2% FBS and 1 mM EDTA (FACS buffer), then plated at 100,000 cells per well. Human mAbs were labeled with the Zenon PE Human IgG labeling kit (Thermo Fisher Cat. #Z25455) according to the manufacturer’s instructions, and 0.25 µg of labeled antibody was added to wells with each transfection condition in triplicate and incubated for 20 min at 4 °C in the dark. Several control wells were stained only with unconjugated Zenon components. The cells were then centrifuged at 500 × *g* for 5 min, washed once with 200 µl of FACS buffer, centrifuged again at 500 × *g* for 5 min, and resuspended in 40 µl FACS buffer. 10 µl from each well was acquired on a BD FACSCelesta, then the median fluorescence intensity (MFI) of the PE-positive cells was calculated for each well using FlowJo v10.8 Software (BD Life Sciences).

For detection of gp42, cells were transfected and plated as described above and incubated with the F-2-1 mAb at 0.3 µg per well for 15 min, then washed with FACS buffer and centrifuged at 500 × *g* for 5 min. Cells were then stained with an anti-mouse PE secondary antibody (BioLegend Cat. #405307) at a 1:200 dilution for 15 min at 4 °C in the dark. Several control wells were stained only with the PE secondary antibody. The cells were then centrifuged at 500 × *g* for 5 min, washed once with 200 µl FACS buffer, centrifuged again at 500 × *g* for 5 min, and fixed in 10% formalin for 10 min at room temperature. Finally, the cells were centrifuged at 500 × *g* for 5 min and resuspended in 40 µl FACS buffer. 10 µl from each well was acquired on a BD FACSCelesta and the median fluorescence intensity (MFI) of the PE-positive cells was calculated for each well using FlowJo v10.8 Software (BD Life Sciences).

### Recombinant proteins

The recombinant gH/gL ectodomain was produced by transfecting pTT3-gH-HIS-AVI and pTT3-gL in 293 6E cells using PEI Max according to the manufacturer’s instructions. Similarly, recombinant gp350 and gp42 were produced by transfecting pTT3-gp350-HIS-AVI and pTT3-gp42-HIS-AVI in 293 6E cells using PEI Max according to the manufacturer’s instructions. All proteins were purified using NiNTA affinity chromatography followed by size exclusion chromatography as previously described^25,34^. Recombinant monoclonal antibodies were produced by co-transfecting heavy and light chain plasmids into 293 6E cells using PEI Max according to the manufacturer’s instructions and purified using Protein A Agarose (Gold Bio Cat. #P-400-5).

### Preparation of EBV reporter viruses

To produce B-cell tropic GFP reporter viruses (B95-8/F), 5 × 10^6^ 293–2089 cells were seeded on a 100 mm tissue culture dish in cRPMI containing 100 μg/ml hygromycin. 48 h later the cells were washed with PBS, the media was replaced with cRPMI without hygromycin, and cells were transfected with 6 μg each of p509 and p2670 expressing BZLF1 and BALF4, respectively, using GeneJuice transfection reagent (SigmaAldrich Cat. #70967)^72,74^. 72 h post transfection, the cell supernatant was collected and centrifuged at 500 × *g* for 3 min to pellet any cell debris, and passed through a 0.8 μm filter. Virions were concentrated 25–50-fold by centrifugation at 25,000 × *g* for 2 h and re-suspended in PBS. The virus was stored at −80 °C and thawed immediately before use.

Epithelial cell tropic virus was produced from Akata-GFP EBV cells suspended at 4 × 10^6^ cells/ml in RPMI containing 1% FBS by adding goat anti-human IgG (Southern Biotech Cat. #2040-01) to a final concentration of 100 μg/ml, and the culture was incubated at 37 °C for 4 h. Cells were then diluted to 2 × 10^6^ cells/ml in RPMI containing 1% FBS and cultured for 72 h. Cultures were centrifuged at 300 × *g* for 10 min to pellet cells and supernatant was passed through a 0.8 μm filter. Bacitracin was added to a final concentration of 100 μg/ml. Virions were concentrated 25× by centrifugation at 25,000 × *g* for 2 h and re-suspended in RPMI containing 100 μg/ml bacitracin. The virus was stored at −80 °C and thawed immediately before use.

### EBV neutralization assay in B cells

EBV neutralization assays were carried out in Raji cells as previously described^75^. In short, serum from mice or human sera was serially diluted in 25 µl cRPMI in triplicate in 96-well round-bottom plates. 12.5 µl of B95-8/F virus diluted to achieve an infection frequency of approximately 1–5% was added and plates were incubated for 1 h at 37 °C. Following the incubation, 12.5 µl of Raji cells at 4 × 10^6^ cells/ml was added to each well and incubated for an hour at 37 °C. Cells were then washed once in cRPMI, re-suspended in fresh cRPMI at 37 °C. 72 h later, cells were fixed in 10% formalin and the percentage of GFP+ Raji cells was determined using a Luminex Guava HT or BDFACS Celesta.

To account for any false positive cells due to auto-fluorescence in the GFP channel, the average %GFP^+^ cells in negative control wells (*n* = 5–10) was subtracted from each well. The infectivity (%GFP^+^) for each well was plotted as a function of the log_10_ of the serum dilution. Serum dilution is reported relative to the final assay volume (50 µl). The neutralization curve was fit using the log (inhibitor) vs response-variable slope (four parameters) analysis in GraphPad Prism 10.0.2 (GraphPad Software). The half maximal inhibitory serum dilution ID_50_ was interpolated from the curve in GraphPad GraphPad Prism 10.0.2. Statistical differences between ID_50_ values for different cohorts were tested at each timepoint by Mann-Whitney test in GraphPad Prism 10.0.2 with no correction for multiplicity.

### EBV neutralization assay in epithelial cells

SVKCR2 cells were seeded at a density of 1.5 × 10^4^ cells per well in a 96 well flat-bottom tissue culture plate. The next day, serum was serially diluted in duplicate wells of a 96 well plate, then Akata-GFP virus was added to each well and incubated for 15 min at 37 °C. The media was then aspirated from the SVKCR2 cells and replaced by the antibody-virus mixture. The plates were incubated at 37 °C for 48 h, then cells were detached from the plate using 0.25% trypsin, transferred to a 96 well round bottom plate, washed twice with PBS, and fixed with 10% formalin. The percentage of GFP+ cells was determined on a BDFACS Celesta and percent neutralization, ID_50_, and statistical differences were determined as in the B cell neutralization assay.

### Immunizations in C57BL/6 mice

Sterile RNA was mixed as a 1:1 volumetric ratio with sterile LION^42^, 40% sucrose, 100 mM citrate, and RNAse free water. Final repRNA concentrations were 1 μg/ml, 10 μg/ml, 100 μg/ml. Immunizations were delivered via split dose intramuscular injection consisting of two 50 μL doses delivered to each rear leg. Blood was collected in serum collection tubes (Sarstedt Cat# 20.1344).

### IgG purification from murine sera

Terminal sera from each group were pooled, diluted in protein G binding buffer (ThermoFisher Cat# 21019), and passed over a column containing 1 ml of protein A/G agarose (ThermoFisher Cat# 20422). The column was then washed three times with five column volumes of binding buffer. Finally, IgG was eluted from the resin in 1 ml fractions using IgG elution buffer (ThermoFisher Cat# 21004) into 0.1 ml of 1 M Tris-HCl, pH 8.0. Fractions were buffer exchanged into PBS, concentrated, passed through a 0.2 µm filter, and quantified by measuring the absorbance at 280 nm using a Nanodrop One (ThermoFisher Cat. #13-400-519).

### Measurement of serum antibody endpoint binding titers by ELISA

30 μl/well of rabbit anti-His tag antibody (SigmaAldrich Cat. #SAB5600227) was adsorbed at a concentration of 0.5 μg/ml on 384 well microplates at 4 °C for 16 h in a solution of 0.1 M NaHCO_3_ pH 9.4–9.6 (coating buffer). The following day, plates were washed four times with 1x PBS and 0.02% Tween 20 (ELISA wash buffer) prior to blocking for 1 h with 90 μl/well of PBS containing 10% non-fat milk and 0.02% Tween 20 (blocking buffer). After blocking, plates were washed four times and 30 μl/well of 2 μg/ml monomeric His-tagged gH/gL diluted in blocking buffer was added to the plate and incubated for 1 hr. 50 ng/well of gp42 or gp350 were adsorbed directly on to 384 well microplates at 4 °C for 16 h in coating buffer, washed four times with ELISA wash buffer and blocked in blocking buffer as above. Plates were washed and sera diluted in blocking buffer was added to the top row of the plate. Three-fold serial dilutions were performed in duplicate followed by a 1 h incubation at 37 °C. Additional control wells containing immobilized gH/gL but no immune sera were included. After washing, a 1:5000 dilution of goat anti-mouse IgG-HRP (SouthernBiotech Cat. #2010-05) in blocking buffer was added to each well and incubated at 37 °C for 1 h. After four washes, 30 μl/well of SureBlue Reserve TMB Microwell Peroxidase substrate (SeraCare Cat. #5120-0081) was added. After 5 min at room temperature, 30 μl/well of 1 N sulfuric acid was added and the A_450_ of each well was read on a Molecular Devices SpectraMax M2 plate reader. The binding threshold was defined as the average plus 10 times the SD of the determined by calculating the average of A_450_ values of the control wells. Endpoint titers were interpolated from the point of the curve that intercepted the binding threshold using the GraphPad Prism 10.0.2 package. Statistical differences between different cohorts were tested at each timepoint by Mann-Whitney test using the GraphPad Prism 10.0.2 package with no correction for multiplicity.

### Measure of competitive binding titers by ELISA

Coating, blocking, and gH/gL immobilization steps were performed as described under “Measurement of serum antibody endpoint binding titers by anti-His capture ELISA.” Following the capture of monomeric gH/gL, equal amounts of sera from each mouse in a group were pooled and diluted in blocking buffer and 2-fold serial dilutions were performed, followed by a 1 h incubation at 37 °C. After washing, monoclonal antibodies AMMO1, CL40, CL59, E1D1, 769B10, 770F7, and 1D8 were added at a concentration that achieves half-maximal binding (EC_50_; pre-determined in the same assay in the absence of competing serum) to each well containing serially diluted pooled sera from each group, followed by a 1 h incubation at 37 °C. After four washes with ELISA washing buffer, a 1:20,000 dilution of goat anti-human IgG-HRP (Jackson ImmunoResearch Cat. # 109-035-088) in blocking buffer was added to each well and incubated at 37 °C for 1 h followed by four washes with ELISA wash buffer. Addition of SureBlue Reserve TMB Microwell Peroxidase substrate, addition of 1 N sulfuric acid, and reading of plates was performed as described above. The average A_450_ values of buffer only control wells were subtracted from each mAb containing well and plotted in GraphPad Prism 10.0.2. A_450_ values were plotted as a function of the log_10_ of the serum dilution. A binding curve was fit using the Sigmoidal, 4PL, X is log(concentration) least squares fit function. Maximum binding was defined as the best-fit value for the top of each curve computed in Prism. A_450_ values at each dilution on the curve were divided by the maximum binding and multiplied by 100 to calculate the % of max binding ([A_450_ at each dilution/max binding] × 100). The titer at which half-maximal binding was observed was interpolated from the binding curve using the GraphPad Prism 10.0.2 package.

### Stimulation of splenocytes

Spleens were harvested from 5 male and 5 female mice immunized with repRNA gHgL or protein monomer gHgL at week 12 post immunization. Splenocytes were isolated by mechanical dissociation in RBC lysis buffer (ThermoFisher Cat. #A1049201) using a 100 μm filter. After dissociation and lysis, cells were washed in FACS buffer once and resuspended in 5 ml FACS buffer. In 96-well plates, splenocytes were plated at a concentration of 2 × 10^6^ cells/well in cRPMI. Cells were stimulated with either cRPMI alone (negative control), 50 μg/ml gH/gL in cRPMI, or 0.5 μg/ml anti-CD3 (ThermoFisher Cat. #14-0037-82) and 0.25 μg/ml anti-CD28 (ThermoFisher Cat. #14-0281-82) (positive control). Cells were incubated at 37 °C, 5% CO_2_ for 24 h prior to start of intracellular staining. Five hours before the end of restimulation 20 μl of brefeldin A (eBioscience Cat. #00-4506-51) at 10 ng/ml and 20 μl 1000× monensin (eBioscience Cat. #00-4505-51) was added to each well.

### Intracellular staining (ICS)

After stimulation, plates were centrifuged at 400 × *g* for 5 min at 8 °C and supernatants were transferred to a new plate and frozen at −20 °C. Cell pellets were resuspended in 200 μl FACS buffer, centrifuged at 400 × *g* for 5 min, and resuspended in 50 μl viability staining mix: 1:500 BV510 live-dead dye (eBioscience Cat. #65-0866-14) and 1:500 Fc Block (Biolegend Cat. #101302) in PBS. Cells were stained on ice in dark for 15 min. 150 μl FACS buffer was added to each well, plates were centrifuged 400 × *g* 5 min, and supernatant removed. Cell pellets were then resuspended in surface staining mix: a 1:200 dilution of the following, anti-mouse CD45 BUV805 (BD Bioscience Cat. #568336), CD3 BUV395 (BD Bioscience Cat. #740268), CD8 BUV737 (BD Bioscience Cat. #612759), and CD4 PerCPCy5.5 (Thermofisher Cat. #45-0042-80) antibodies in FACS buffer. Cells were stained on ice in dark 30 min. After staining, cells were resuspended in 150 μl FACS buffer and washed once in 200 μl FACS buffer. Cells were then fixed and permeabilized for 20 min on ice using 100 μl 1X CytoFix solution (BD Bioscience Cat. #554714). Plates were then washed twice in 1X CytoPerm Wash Buffer (BD Bioscience Cat. #554714). ICS was then done by resuspension in 50 μl/well ICS mix: in CytoPerm wash buffer, a 1:200 dilution anti-mouse IFN-ɣ AF488 (Biolegend Cat. #505815). Cells were stained on ice in dark for 30 min. Cells washed twice in CytoPerm wash buffer and resuspended in FACS buffer for acquisition. Samples were acquired on BD Fortessa X50 cytometer. The frequency of IFNɣ+ cells in the Lymphocyte/Singlet/Live/CD45^+^/CD4^+^ or CD8^+^ population was determined for each sample. The frequency of CD4^+^ or CD8^+^ T cells expressing IFNɣ from baseline cRPMI stimulation was subtracted from the final reported values.

### EBV infection in humanized mice

Twenty-five six-week-old NSG mice were irradiated (275R of total body irradiation) and received 1 × 10^6^ CD34^+^ huPBSC in 200 µl PBS through i.v. injection. Eight weeks later, successful human cell engraftment was confirmed by the presence of human CD45^+^ cells in peripheral blood by flow cytometry. Using 50 µl blood, RBCs were lysed and cells were stained using a BV510 viability dye, and the following antibodies at a 1:100 dilution unless otherwise noted: hCD45 FITC (eBioscience Cat. #5010066), mCD45 APC (eBioscience Cat. #17-0451-82) (1:500 dilution), hCD33 PE (BD Bioscience Cat. #555450), hCD19 BV711 (Biolegend Cat.# 302246), hCD4 AF700 (eBioscience Cat. #56-0048-82) and hCD8 BV421 (BD Bioscience Cat. #562429). Cells were stained for 30 min on ice, washed twice in FACS buffer, fixed in 200 µl of 10% formalin 15 min on ice, washed and resuspended in 200 µl FACS buffer for acquisition and analyzed on a BDFACS Celesta. 10 weeks post-engraftment, 500 µg of experimental or control antibodies were injected per humanized NSG mouse via intraperitoneal injection (i.p.). 24 h later, mice were bled in the left eye to confirm passive transfer of IgG, and received a dose of EBV B95.8/F67 equivalent to 33,000 Raji infectious units as determined by infection of Raji cells via intravenous injection in the right eye. Each group of mice receiving the same IgG preparation and/or EBV were housed separately from each other. Mice were weighed three times weekly. Beginning at 2 weeks post-infection, peripheral blood samples were collected to measure the presence of EBV DNA in whole blood. Twelve weeks post-challenge, or until mice lost 20% of their starting weight, mice were euthanized. Spleens were photographed and weighed, then DNA was extracted from splenocytes utilizing the DNeasy Blood & Tissue Kit (QIAGEN) and according to the manufacturer’s instructions for subsequent viral load analysis.

### Measurement of total serum IgG in huCD34+ engrafted NSG mice

Serum was serially diluted in ELISA coating buffer in duplicate and incubated on 384-well microplates at 4 °C for 16 h. At least 10 additional control wells were included that contained only coating buffer and no sera. The next day, plates were washed 4× with ELISA wash buffer prior to blocking for 1 h with 100 μl/well of ELISA blocking buffer. After blocking, plates were washed and a 1:4000 dilution of goat anti-mouse IgG Human ads-HRP in ELISA blocking buffer was added to each well and incubated 1 hr at 37 °C. Plates were washed and addition of SureBlue Reserve TMB Microwell Peroxidase substrate, addition of 1 N sulfuric acid, and reading of plates was performed as described above. The average A450 values of buffer only control wells were subtracted from each serum containing well and plotted in GraphPad Prism 10.0.2. A450 values were plotted as a function of the log10 of the serum dilution. A binding curve was fit using the Sigmoidal, 4PL, X is log(concentration) least squares fit function. The binding threshold was determined as in “Measurement of sera antibody endpoint binding titers by anti-His capture ELISA”.

### Quantitative PCR analysis EBV DNA in huCD34 engrafted mice

A primer-probe mix specific for the EBV BALF5 gene^76^ was used to quantify EBV in DNA extracted from blood or spleen in hCD34 engrafted NSG recipient mice at the time points described. Each 25 μl qPCR reaction contained 12.5 μl QuantiTect Probe PCR Master Mix (QIAGEN), 600 nM of each primer and 300 nM of FAM-labeled probe (IDT), 1.25 μl of a TaqMan VIC-labeled RNase-P primer probe mix (Fisher Sci Cat. #4316844). For analysis of splenocytes, reactions contained 1 μg DNA extracted from splenocytes as template. To analyze EBV in peripheral blood, 50 μl of blood collected via cardiac puncture or retro-orbital bleed DNA extracted using the DNeasy Blood and Tissue Kit (QIAGEN) and eluted in 50 μl of Buffer AE (QIAGEN). 10 μl of extracted DNA was used as template in qPCR. Reactions were heated to 95 °C for 15 min to activate DNA polymerase followed by 50 cycles of 95 °C for 15 s 60 °C for 60 s, on an Applied Biosystems QuantStudio 7 Flex Real-Time PCR System. Synthetic DNA fragments containing the BALF5 target gene as well as flanking genomic regions were synthesized as double stranded DNA gBlocks (IDT), and were used to generate a standard curve with known gene copy numbers ranging from 10^7^ to 10^0^ copies/μl. The copy number in extracted DNA was determined by interpolating from the standard curve. Serial dilutions of reference standard were used to experimentally determine a limit of detection of 6.25 copies, which corresponds to the amount of template that can be detected in >95% of reactions. For graphical purposes, samples with no amplification or those yielding values below the limit of detection were assigned a value of 0.625 copies.

### Statistical analysis

Mann-Whitney tests were used to compare the distribution of outcomes between the pairs of groups considered. Normal distribution was not assumed because of sample size. *p* values < 0.05 considered significant. Immunogenicity was compared across each pair of treatment groups; for spleen weights and viral DNA copies, each group was compared to the infected control. For survival data, significant differences were determined using Log-rank Mantel-Cox test. Statistical tests were performed using GraphPad Prism version 10 or higher.

### Inclusion and ethics statement

All co-authors on this study have met the criteria for authorship outlined by Nature Portfolio journals, and the roles and responsibilities of each author were agreed among researchers. All authors agree to the authors’ contribution statement. This research was not limited by the location of the researchers, and all where appropriate has been reviewed and approved by relevant institutional committees as noted in the methods. This work does not result in incrimination, stigmatization, or discrimination and presents no safety risk to researchers. Local and regional research relevant to our study was taken into account in citations.

### Materials availability

All materials generated herein are available upon request under an MTA from the corresponding author (amcguire@fredhutch.org). The pTT3 plasmids are used under license from the National Research Council of Canada.

### Reporting summary

Further information on research design is available in the Nature Research Reporting Summary linked to this article.

### Supplementary information

Supplementary Information

Reporting Summary

## Data Availability

No data with mandated deposition was generated as a result of this study. Other data included in this study will be made available upon request to amcguire@fredhutch.org.
