# Supplementary material for: A gH/gL-encoding replicon vaccine elicits neutralizing antibodies that protect humanized mice against EBV challenge
Source: NPJ Vaccines. 2024 Jun 26;9:120. doi: 10.1038/s41541-024-00907-y (PMC11208421; doi:10.1038/s41541-024-00907-y)
Supplement: Supplementary file 1 — Supplementary Information [file 41541_2024_907_MOESM1_ESM.pdf]

Supplementary Table 1: Statistical analyses for data shown in Figure 2.

**ELISA**	**0**	**2**	**4**	**6**	**8**	**10**	**12**	**14**	**18**	**26**	**32**
1 µg/1 µg	ND	0.004	0.0286	0.1143	0.0162	0.004	0.0286	0.0286	0.004	ND	ND
0.1 µg/ 0.1 µg	ND	0.0002	0.004	0.004	0.0002	0.0006	0.004	0.004	0.0002	ND	ND
10 µg/ 0.1 µg	ND	ND	ND	ND	0.2844	0.0016	0.0159	0.0159	NA	0.1905	0.1111
10 µg/ 1 µg	ND	ND	ND	ND	0.2844	0.0016	0.0159	0.0159	NA	0.1111	0.0571
0.1 µg/ 10 µg	ND	ND	ND	ND	0.004	0.004	0.2	0.0286	NA	0.4857	0.6286
**Epithelial Cell Neutralization**	**0**	**2**	**4**	**6**	**8**	**10**	**12**	**14**	**18**	**26**	**32**
1 µg/1 µg	ND	0.004	0.2	0.2	0.0162	0.004	0.0286	0.0286	0.004	ND	ND
0.1 µg/ 0.1 µg	ND	0.004	0.1143	0.1143	0.0081	0.0081	0.0286	0.0286	0.004	ND	ND
10 µg/ 0.1 µg	ND	ND	ND	ND	0.0451	0.0016	0.0159	0.0159	ND	0.0159	0.0286
10 µg/ 1 µg	ND	ND	ND	ND	0.0109	0.0016	0.0159	0.0159	ND	0.0159	0.0159
0.1 µg/ 10 µg	ND	ND	ND	ND	0.004	0.004	0.0286	0.1143	ND	0.0286	0.0571
**B Cell Neutralization**	**0**	**2**	**4**	**6**	**8**	**10**	**12**	**14**	**18**	**26**	**32**
1 µg/1 µg	ND	0.2081	>0.9999	0.1429	0.0485	0.004	0.0286	0.0286	0.004	ND	ND
0.1 µg/ 0.1 µg	ND	0.2081	>0.9999	0.1429	0.0485	0.004	0.0286	0.0286	0.004	ND	ND
10 µg/ 0.1 µg	ND	ND	ND	ND	0.2059	0.0016	0.0556	0.0159	ND	0.0286	0.0286
10 µg/ 1 µg	ND	ND	ND	ND	0.2059	0.0016	0.0556	0.0159	ND	0.0159	0.0286
0.1 µg/ 10 µg	ND	ND	ND	ND	0.0264	0.004	0.0159	0.0286	ND	0.0286	0.0571

Titers at individual timepoints were compared to the 10 µg/10 µg LION/repRNA gH/gL group and summarized above. Significance was calculated by Mann-Whitney test. ND – Not Done. Two-tailed p values shown in table.

Supplementary Table 2: Statistical analyses for data shown in Figure 4.

**ELISA**	**0**	**2**	**4**	**6**	**8**	**10**	**12**	**14**	**18**	**22/23**	**32/34**
4-mer	ND	0.3543	>0.9999	>0.9999	0.7242	0.3543	0.4127	0.0635	0.3543	0.0635	0.0317
C4b (7-mer)	ND	0.0653	0.4127	0.1905	0.0451	0.1709	0.2857	0.1905	0.6216	0.0635	0.0317
I3 (60-mer)	ND	0.7242	>0.9999	0.5556	0.2844	0.0031	0.0317	0.0159	0.004	0.0286	0.0286
**Epithelial Cell Neutralization**	**0**	**2**	**4**	**6**	**8**	**10**	**12**	**14**	**18**	**22/23**	**32/34**
4-mer	ND	0.0653	0.1111	0.0317	0.1709	0.0295	0.0317	0.5556	0.0186	0.0159	0.0159
C4b (7-mer)	ND	0.2844	0.2857	0.0079	0.0451	0.0016	0.0159	0.0159	0.0062	0.0159	0.0159
I3 (60-mer)	ND	0.0186	0.0571	0.0159	0.0031	0.0031	0.0159	0.0159	0.0162	0.0571	0.0286
**B Cell Neutralization**	**0**	**2**	**4**	**6**	**8**	**10**	**12**	**14**	**18**	**22/23**	**32/34**
4-mer	ND	0.1608	>0.9999	0.127	0.0264	0.0016	0.0317	0.0317	0.3543	0.0159	0.0159
C4b (7-mer)	ND	0.1608	0.4444	0.9762	0.0505	0.0016	0.0159	0.0317	0.0016	0.0635	0.0159
I3 (60-mer)	ND	0.1608	0.4444	0.0476	0.0264	0.0016	0.0159	0.0159	0.004	0.0286	0.0286

Titers at individual timepoints were compared to the 10 µg/10 µg LION/repRNA encoded full length gH/gL group and summarized above. Significance was calculated by Mann-Whitney test. ND – Not Done, two-tailed p values shown in table. Week 22 post-immunization of the 10 µg/10 µg LION/repRNA encoded full-length gH/gL was compared to week 23 of the multimer groups, and week 32 compared to week 34.

Supplementary Table 3: Summary of results from EBV challenge of humanized mice after passive transfer of vaccine elicited IgG.

Mouse	Group	Post-transfer IgG endpoint	Post-transfer gHgL binding endpoint	Viral titer in blood at euthanasia (viral copies/µl blood)	Week first positive for BALF5	Survival (weeks)	Spleen viral load	Spleen weight (mg)
846	PBS	N/A	N/A	0.625	N/A	12	0.625	145
847	PBS	N/A	N/A	0.625	N/A	12	0.625	60
848	PBS	N/A	N/A	0.625	N/A	12	2.274	61
849	PBS	N/A	N/A	0.625	N/A	12	0.895	58
836	Protein	1.012E+06	2.278E+04	0.625	N/A	12	3.377	88
837	Protein	2.273E+05	7.164E+04	30.936	12	12	72.813	240
838	Protein	1.446E+05	3.487E+04	1.510	7	12	106.522	45
839	Protein	1.291E+05	3.778E+04	2357.039	7	8	502.856	194
840	Protein	1.271E+05	3.356E+03	4855.516	9	9	815.711	259
831	repRNA	2.679E+05	3.905E+05	0.625	N/A	12	0.724	65
832	repRNA	1.986E+05	3.218E+05	0.625	N/A	12	0.678	68
833	repRNA	3.011E+05	3.763E+05	0.625	4	12	2.119	81
834	repRNA	2.192E+05	3.905E+05	0.625	8	12	0.625	31
851	Control IgG	1.610E+05	N/A	10.727	5	5	207.571	200
852	Control IgG	1.116E+05	N/A	8673.307	6	8	1926.007	301
853	Control IgG	1.942E+05	N/A	9.461	8	8	191.058	133
854	Control IgG	2.049E+05	N/A	202.075	7	8	542.984	245
856	Control IgG	2.408E+05	N/A	156.191	5	5	1069.962	166

Related to Figure 5. Summary of transferred IgG, viral titers, spleen weight, and survival for each individual mouse.

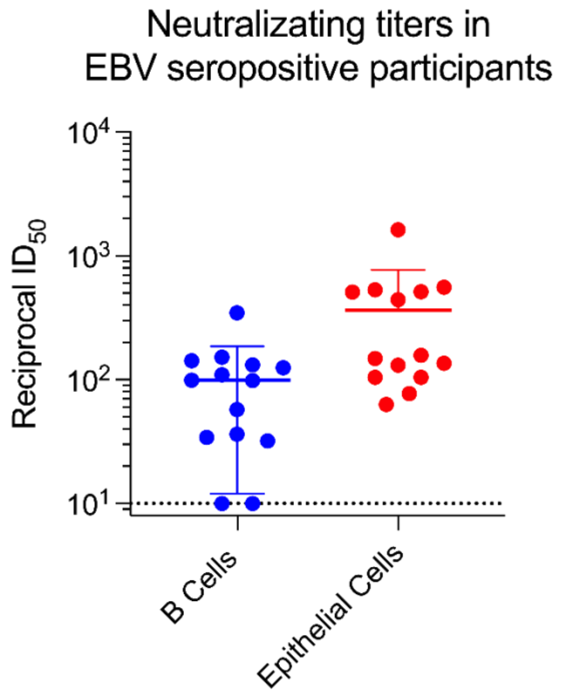

Supplementary Figure 1. The ability of sera from EBV seropositive donors to neutralize EBV infection of B cells or epithelial cells as indicated. Each dot represents the reciprocal half-maximal inhibitory concentration of an individual donor (n=14) measured in duplicate. Bars represent the mean. Dashed line indicates the lowest serum dilution tested.

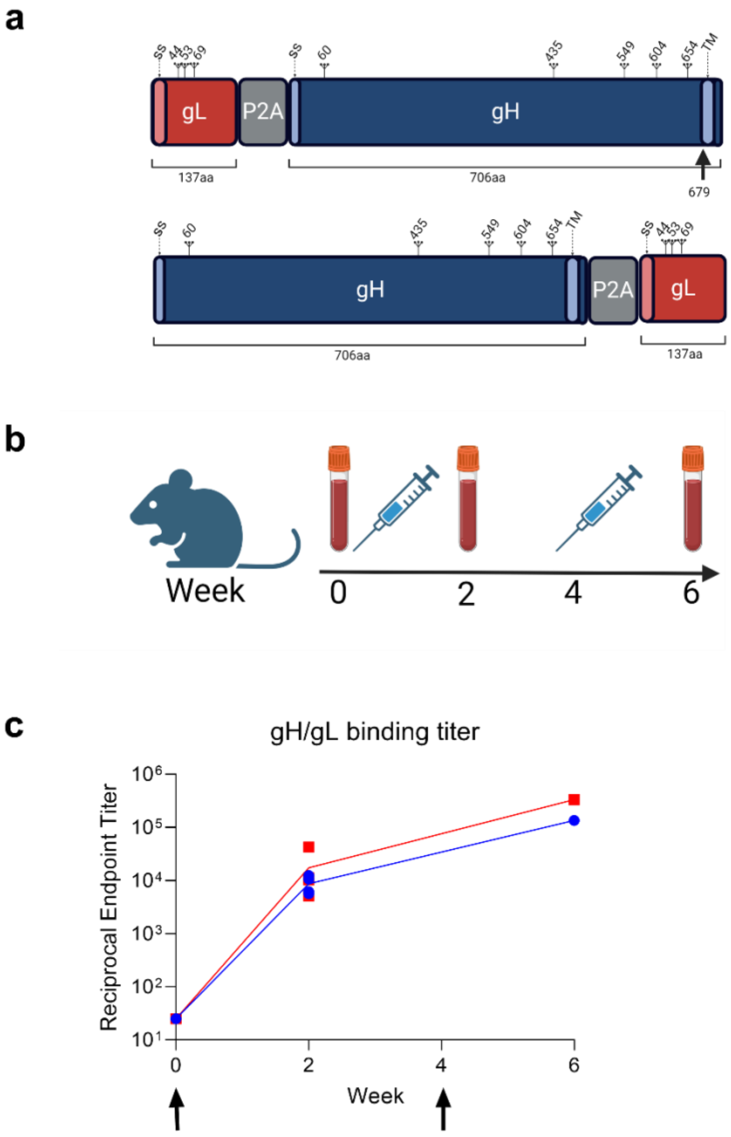

Supplementary Figure 2. Immunogenicity of repRNAs encoding EBV gH/gL in two orientations. (**a**) schematic depicting the two repRNA inserts encoding gH and gL separated by a P2A peptide with the order swapped. The boundary of the gH ectodomain at amino acid 679 is indicated on the top representation. (**b**) Immunization schedule in C57BL/6 mice. (**c**) Reciprocal endpoint gH/gL binding titers were measured in plasma by ELISA. Each dot represents an individual mouse (n=4) at weeks 0 and 2, or pooled sera from the same 4 mice at week 6. The lines connect the mean (or value of pooled sera) across the tested timepoints. Arrows indicate time of immunization. Panels **a** and **b** created with BioRender.com.

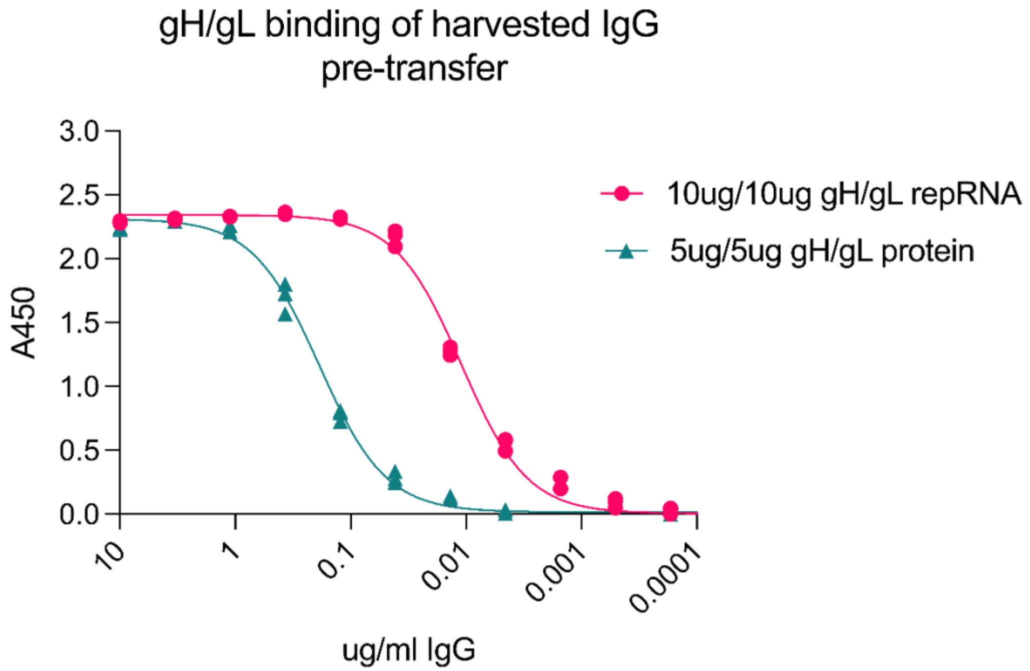

Supplementary Figure 3: gH/gL binding of purified IgG after two immunizations with full length gH/gL encoded by repRNA or the recombinant gH/gL ectodomain as indicated. gH/gL binding was measured for each purified IgG in triplicate. The purified IgG was used for transfer experiments in Figure 5.

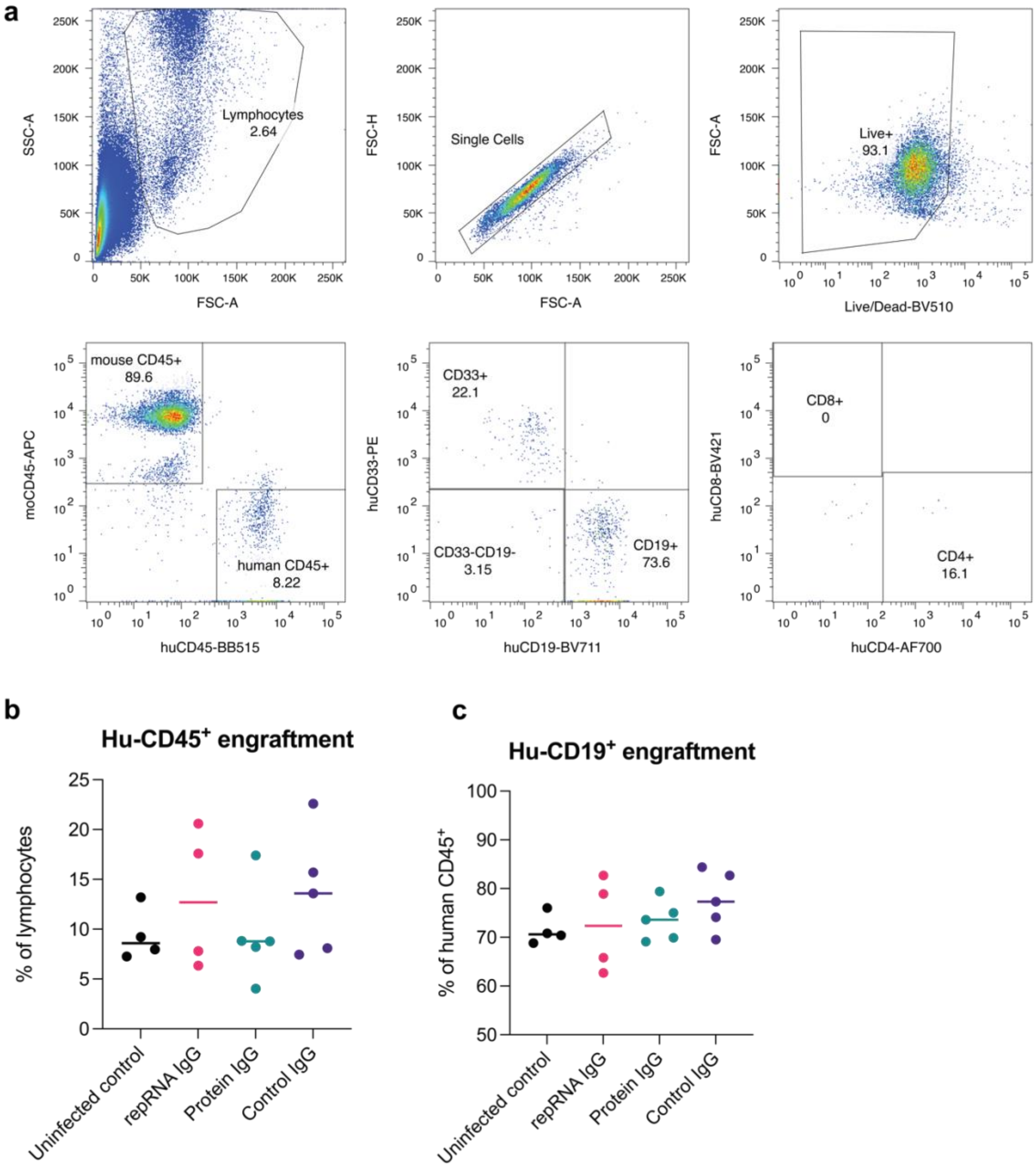

Supplementary Figure 4: Cell engraftment in humanized mice used in Figure 5. (**a**) Gating strategy to determine human CD45$^+$ cells within live single cell lymphocyte population as well as CD19$^+$ B cells within human CD45$^+$ cells. Representative plots from one mouse shown. (**b**-**c**) Frequency of human CD45$^+$ lymphocytes (**b**) and human CD19$^+$ B cells (**c**) in the mice assigned to the various treatment groups. n=5 for repRNA, uninfected control, and control IgG groups and n=4 for protein IgG group.

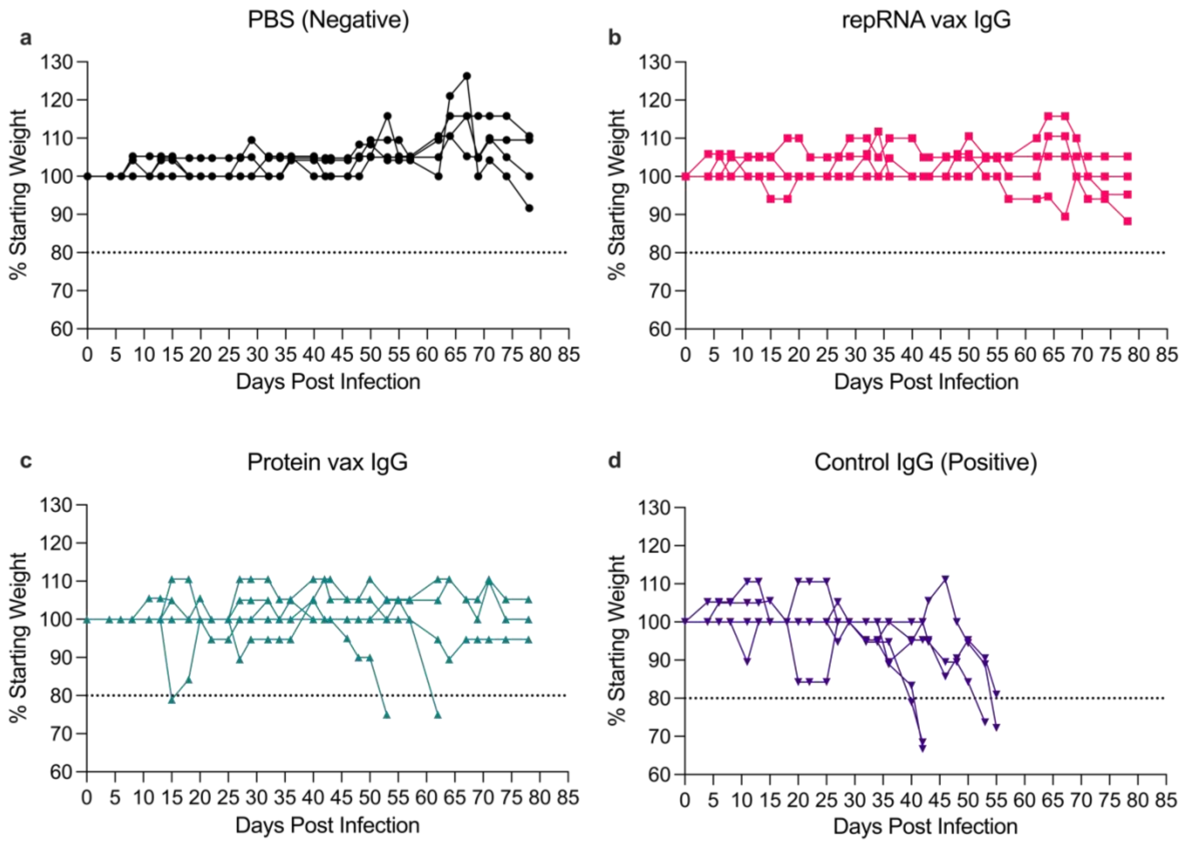

Supplementary Figure 5: Weight of mice from Figure 5 after EBV challenge. The percent of the starting weight over time for (**a**) uninfected control mice treated with PBS, (**b**) mice given IgG elicited by LION/repRNA-gH/gL prior to EBV challenge, (**c**) mice given IgG elicited by protein vaccination prior to challenge, and (**d**) mice given control IgG prior to challenge (positive control). Dashed line indicates threshold for humane endpoint.

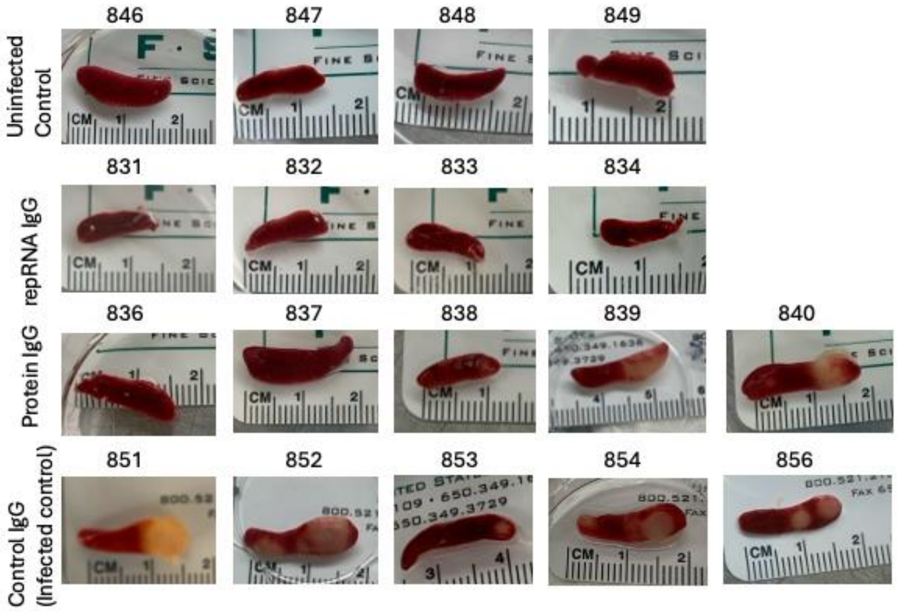

Supplementary Figure 6: Spleens from individual animals from Figure 5 collected at time of euthanasia.

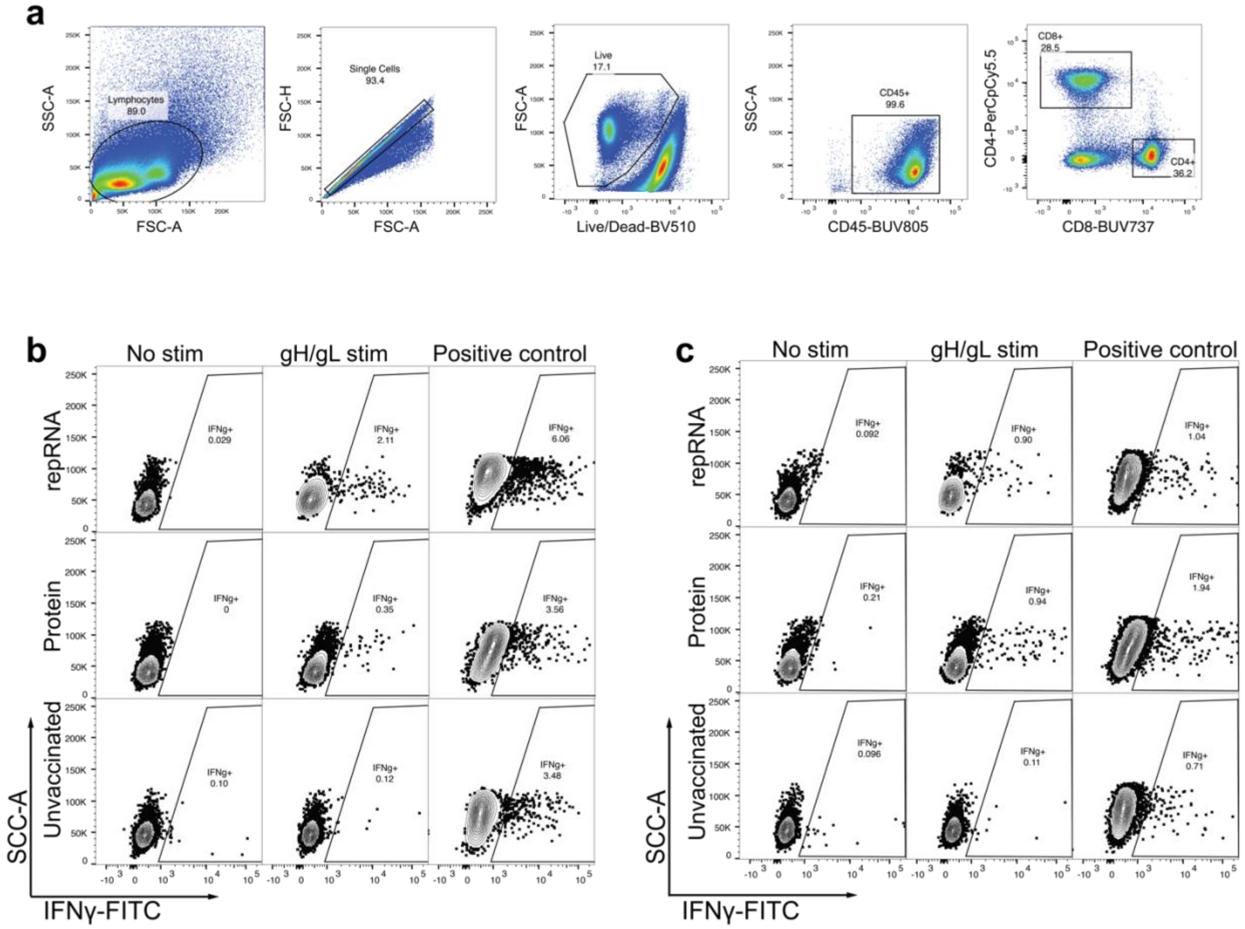

Supplementary Figure 7: Gating strategy for evaluating IFNɣ$^+$ CD4$^+$ and CD8$^+$ T cells. Related to figure 6. (**a**) Gating strategy for CD4$^+$ and CD8$^+$ T cell populations. Representative data shown from one mouse. (**b**) IFNɣ$^+$ CD8$^+$ T cell populations for a representative repRNA immunized, protein immunized, and unimmunized animal after media only stimulation (negative control), gH/gL stimulation, and positive control simulation (anti-CD3/CD28) of splenocytes as indicated. (**c**) IFNɣ$^+$ staining within CD4$^+$ T cell populations for a representative repRNA immunized, protein immunized, and unimmunized animal after media only stimulation (negative control), gH/gL stimulation, and positive control simulation (anti-CD3/CD28) of splenocytes as indicated. Data from one representative mouse per immunization group is shown.
