# Supplementary material for: A gH/gL-encoding replicon vaccine elicits neutralizing antibodies that protect humanized mice against EBV challenge
Source: NPJ Vaccines. 2024 Jun 26;9:120. doi: 10.1038/s41541-024-00907-y (PMC11208421; doi:10.1038/s41541-024-00907-y)
Supplement: Supplementary file 2 — Reporting Summary [file 41541_2024_907_MOESM2_ESM.pdf]

nature portfolio

Corresponding author(s): Andrew McGuire

Last updated by author(s): May 9, 2024

Reporting Summary

Nature Portfolio wishes to improve the reproducibility of the work that we publish. This form provides structure for consistency and transparency in reporting. For further information on Nature Portfolio policies, see our Editorial Policies and the Editorial Policy Checklist.

Statistics

For all statistical analyses, confirm that the following items are present in the figure legend, table legend, main text, or Methods section.

n/a	Confirmed	
☐	☒	The exact sample size (*n*) for each experimental group/condition, given as a discrete number and unit of measurement
☐	☒	A statement on whether measurements were taken from distinct samples or whether the same sample was measured repeatedly
☐	☒	The statistical test(s) used AND whether they are one- or two-sided<br>*Only common tests should be described solely by name; describe more complex techniques in the Methods section.*
☒	☐	A description of all covariates tested
☐	☒	A description of any assumptions or corrections, such as tests of normality and adjustment for multiple comparisons
☐	☒	A full description of the statistical parameters including central tendency (e.g. means) or other basic estimates (e.g. regression coefficient) AND variation (e.g. standard deviation) or associated estimates of uncertainty (e.g. confidence intervals)
☒	☐	For null hypothesis testing, the test statistic (e.g. *F*, *t*, *r*) with confidence intervals, effect sizes, degrees of freedom and *P* value noted<br>*Give P values as exact values whenever suitable.*
☒	☐	For Bayesian analysis, information on the choice of priors and Markov chain Monte Carlo settings
☒	☐	For hierarchical and complex designs, identification of the appropriate level for tests and full reporting of outcomes
☒	☐	Estimates of effect sizes (e.g. Cohen's *d*, Pearson's *r*), indicating how they were calculated

Our web collection on statistics for biologists contains articles on many of the points above.

Software and code

Policy information about availability of computer code

Data collection	No Software was used.
Data analysis	Statistical tests were performed using GraphPad Prism version 10 or higher.

For manuscripts utilizing custom algorithms or software that are central to the research but not yet described in published literature, software must be made available to editors and reviewers. We strongly encourage code deposition in a community repository (e.g. GitHub). See the Nature Portfolio guidelines for submitting code & software for further information.

Data

Policy information about availability of data

All manuscripts must include a data availability statement. This statement should provide the following information, where applicable:

- Accession codes, unique identifiers, or web links for publicly available datasets
- A description of any restrictions on data availability
- For clinical datasets or third party data, please ensure that the statement adheres to our policy

No data with mandated deposition was generated as a result of this study. Other data included in this study will be made available upon request to amcguire@fredhutch.org.

Research involving human participants, their data, or biological material

Policy information about studies with human participants or human data. See also policy information about sex, gender (identity/presentation), and sexual orientation and race, ethnicity and racism.

Reporting on sex and gender	Donors were selected randomly and no considerations were made for age or sex.
Reporting on race, ethnicity, or other socially relevant groupings	Donors were selected randomly and no considerations were made for age or sex.
Population characteristics	Donors were selected randomly and no considerations were made for age or sex.
Recruitment	Donors were selected randomly and no considerations were made for age or sex.
Ethics oversight	Fred Hutch Cancer Center Institutional Animal Care and Use Committee and Institutional Review Boards

Note that full information on the approval of the study protocol must also be provided in the manuscript.

Field-specific reporting

Please select the one below that is the best fit for your research. If you are not sure, read the appropriate sections before making your selection.

☒ Life sciences ☐ Behavioural & social sciences ☐ Ecological, evolutionary & environmental sciences

For a reference copy of the document with all sections, see nature.com/documents/nr-reporting-summary-flat.pdf

Life sciences study design

All studies must disclose on these points even when the disclosure is negative.

Sample size	Groups of 4-10 mice were used for comparative immunogenicity studies. Group sizes used in these studies are comparable to similar studies previously reported
Data exclusions	No Data were excluded from the analyses.
Replication	Multiple mice (n≥4) were included in each experimental group. Independent replicates of serological assays and biochemical assays were performed in duplicate or triplicate. At least 2 independent replicates were performed.
Randomization	Equal numbers of male and female mice were used.
Blinding	No blinding was used.

Reporting for specific materials, systems and methods

We require information from authors about some types of materials, experimental systems and methods used in many studies. Here, indicate whether each material, system or method listed is relevant to your study. If you are not sure if a list item applies to your research, read the appropriate section before selecting a response.

Materials & experimental systems

n/a	Involved in the study	
☐	☒	Antibodies
☒	☐	Eukaryotic cell lines
☒	☐	Palaeontology and archaeology
☐	☒	Animals and other organisms
☒	☐	Clinical data
☒	☐	Dual use research of concern
☒	☐	Plants

Methods

n/a	Involved in the study	
☒	☐	ChIP-seq
☐	☒	Flow cytometry
☒	☐	MRI-based neuroimaging

Antibodies

Antibodies used	anti-mouse PE secondary antibody (BioLegend Cat. #405307), rabbit anti-His tag antibody (SigmaAldrich Cat. #SAB5600227),anti-mouse IgG-HRP (SouthernBiotech Cat. #2010-05) ,goat anti-human IgG-HRP (Jackson ImmunoResearch Cat. # 109-035-088) ,anti-CD3 (ThermoFisher Cat. #14-0037-82) ,anti-CD28 (ThermoFisher Cat. #14-0281-82) ,CD45 BUV805 (BD Bioscience Cat. #568336), CD3

	BUV395 (BD Bioscience Cat. #740268), CD8 BUV737 (BD Bioscience Cat. #612759), and CD4 PerCPCy5.5 (Thermofisher Cat. #45-0042-80) ,anti-mouse IFN-ɣ AF488 (Biolegend Cat. #505815),hCD45 FITC (eBioscience Cat. #5010066), mCD45 APC (eBioscience Cat. #17-0451-82) ,), hCD33 PE (BD Bioscience Cat. #555450), hCD19 BV711 (Biolegend Cat.# 302246), hCD4 AF700 (eBioscience Cat. #56-0048-82),hCD8 BV421 (BD Bioscience Cat. #562429).
Validation	Antibodies were used in accordance with the manufactuer's website, assuming species and target specificity was validated by manufacturer.

Animals and other research organisms

Policy information about studies involving animals; ARRIVE guidelines recommended for reporting animal research, and Sex and Gender in Research

Laboratory animals	Comparative immunogenicity studies were performed in groups of 4 or 5 C57BL/6 mice between 7 and 10 weeks of age.Mice were purchased from the Jackson Laboratory. Six week old NSG female mice were used for humanized mouse studies.
Wild animals	The study did not use wild animals.
Reporting on sex	Equal numbers of male and female mice were used for comparative immunogenicity studies. Female NSG mice were used for huCD34 engraftment. Human serum was randomly selected for assays with no consideration for sex. Data was not analyzed with considerations for sex.
Field-collected samples	The study did not use field collected samples.
Ethics oversight	Fred Hutch Cancer Center Institutional Animal Care and Use Committee .

Note that full information on the approval of the study protocol must also be provided in the manuscript.

Plants

Seed stocks	N/A
Novel plant genotypes	N/A
Authentication	N/A

Flow Cytometry

Plots

Confirm that:

☒ The axis labels state the marker and fluorochrome used (e.g. CD4-FITC).

☒ The axis scales are clearly visible. Include numbers along axes only for bottom left plot of group (a 'group' is an analysis of identical markers).

☒ All plots are contour plots with outliers or pseudocolor plots.

☒ A numerical value for number of cells or percentage (with statistics) is provided.

Methodology

Sample preparation	PBMC from whole blood collected from humanized mice. Splenocytes were mechanically disrupted from C57/Bl6 mice. Cultured cells were transfected and stained with flourescent antibodies or infected with fluorescent reporter virus.
Instrument	BD FACSCelesta Special Order Research Product, Luminex Guava HT
Software	FlowJo v10.8 Software (BD Life Sciences).
Cell population abundance	No sorting was performed.
Gating strategy	Gating strategy is provided in supplementary information

☒ Tick this box to confirm that a figure exemplifying the gating strategy is provided in the Supplementary Information.
